# Recombination and structural variation in a large 8-founder wheat MAGIC population

**DOI:** 10.1093/g3journal/jkaf037

**Published:** 2025-02-21

**Authors:** Rohan Shah, B Emma Huang, Alex Whan, Nick S Fradgley, Marcus Newberry, Klara Verbyla, Matthew K Morell, Colin R Cavanagh

**Affiliations:** CSIRO, Agriculture and Food, Canberra, ACT 2601, Australia; CSIRO, Agriculture and Food, Canberra, ACT 2601, Australia; CSIRO, Agriculture and Food, Canberra, ACT 2601, Australia; CSIRO, Agriculture and Food, Canberra, ACT 2601, Australia; CSIRO, Agriculture and Food, Canberra, ACT 2601, Australia; CSIRO, Agriculture and Food, Canberra, ACT 2601, Australia; CSIRO, Agriculture and Food, Canberra, ACT 2601, Australia; CSIRO, Agriculture and Food, Canberra, ACT 2601, Australia

**Keywords:** Multiparent Advanced Generation Inter-Cross (MAGIC), segregation distortion, bread wheat, multiparental populations, recombination, MPP

## Abstract

Identifying the genetic architecture of complex traits requires access to populations with sufficient genetic diversity and recombination. Multiparent Advanced Generation InterCross (MAGIC) populations are a powerful resource due to their balanced population structure, allelic diversity, and enhanced recombination. However, implementing a MAGIC population in complex polyploids such as wheat is challenging, as wheat harbors many introgressions, inversions, and other genetic factors that interfere with linkage mapping. By utilizing a comprehensive crossing strategy, additional rounds of mixing, and novel genotype calling approaches, we developed a bread wheat 8-parent MAGIC population of over 3,000 genotyped recombinant inbred lines derived from 2,151 distinct crosses. This effort resulted in a dense genetic map covering the complete genome. Further rounds of intercrossing led to increased recombination in inbred lines, as expected. We identified structural variation highlighted by segregation distortion, along with epistatic allelic interactions between specific founders. We report on a novel and effective resource for genomic and trait exploration in hexaploid wheat, capable of detecting small genetic effects and epistatic interactions due to the high level of recombination and large number of lines. The interactions and genetic effects identified provide a basis for ongoing research to understand the basis of allelic frequencies across the genome, particularly where economically important loci are involved.

## Introduction

Due to the large genome size of bread wheat and its hexaploid nature, powerful genetic resources are required to understand the underlying genetic mechanisms for a wide variety of phenotypes. While directed biparental populations and association mapping panels have been developed for this purpose, multiparental populations offer a novel opportunity to dissect genomic structure by combining strengths of both prior approaches, leveraging the specifity of designed crosses with the increased recombination and diversity of more founder lines (Scott *et al.* 2020). In particular, Multiparent Advanced Generation InterCrosses (MAGIC) mix the genomes of several diverse founders through multiple generations of intercrossing and selfing or double haploidy, to generate a large population of immortalized lines.

MAGIC populations have been developed as genetic resource panels in a number of species. For review, see (Scott *et al.* 2020) and (Samantara *et al.* 2021). The first plant MAGIC population was developed in *Arabidopsis thaliana* (Kover *et al.* 2009), and since then populations have been developed in crops including barley (Mathew *et al.* 2018), chickpea (Gaur *et al.* 2012), maize (Dell’Acqua *et al.* 2015), rice (Bandillo *et al.* 2013), ryegrass (Begheyn *et al.* 2018), sorghum (Ongom and Ejeta 2018), and tomato (Pascual *et al.* 2015). More recently, an 8-parent winter wheat population was described (Stadlmeier *et al.* 2018) with a dramatically simplified crossing structure and relatively small size of 394 Recombinant Inbred Lines (RILs). Neither this population nor those in other crops explore the full range of potential intercrosses possible in the early stages of MAGIC designs, and only a few of them have genotyped more than 1,000 lines. The four-parent spring wheat MAGIC population (Huang *et al.* 2012) consists of nearly 1,500 RILs genotyped at high density, while the 8-parent winter wheat MAGIC (Mackay *et al.* 2014) consists of 1,091 F7RILs, of which 720 have been genotyped with the 90 K SNP chip (Wang *et al.* 2014). The wealth of genetic data and genetic diversity facilitates the use of these populations for uncovering genomic structures.

Thus far, MAGIC populations have been used primarily for linkage map construction and quantitative trait loci (QTL) mapping. High-density linkage maps have been constructed in wheat (Huang *et al.* 2012; Cavanagh *et al.* 2013; Gardner *et al.* 2016; Sannemann *et al.* 2018; Fradgley *et al.* 2023), durum wheat (Milner *et al.* 2016), barley (Sannemann *et al.* 2015), and tomato (Pascual *et al.* 2015), and validated against consensus and physical maps. The diversity and resolution of MAGIC populations enables more precise mapping of more markers than in previous populations. The resulting maps provide greater resolution in QTL mapping, which has been performed in all of the crops previously mentioned, both for proof of concept (Kover *et al.* 2009; Huang *et al.* 2012; Sannemann *et al.* 2015) and discovery of novel loci (Rebetzke *et al.* 2014; Barrero *et al.* 2015; Scott *et al.* 2021).

The rich genomic information contained in these populations enables investigation of genomic structure at a level of detail not possible in biparental populations. Multiparental populations have been used to demonstrate widespread genetic incompatibilities in Drosophila, Arabidopsis, and maize (Corbett-Detig *et al.* 2013), and characterize regions associated with maternal transmission ratio distortion in mice (Didion *et al.* 2015). Gardner *et al.* (2016) and Scott *et al.* (2021) also identified widespread segregation distortion and introgressions in 8 and 16 founder wheat MAGIC populations, respectively. The large nonrecombining blocks present in the wheat genome (Choulet *et al.* 2014) presents particular challenges for introgression and selection of novel genetic effects in breeding programmes due to linkage drag (Hao *et al.* 2020). Controlled recombination has been proposed as a solution to minimize negative effects of deleterious linked genes and unlock variation for breeding while positive linkage of beneficial haplotypes has also been shown to aid longer term genetic gain in wheat breeding (Fradgley *et al.* 2019; Taagen *et al.* 2022).

The production of a high-quality reference sequence for bread wheat has been a challenging problem, due to its large genome size and three separate subgenomes. Significant advancements have been made recently in the availability of reference genome sequence (Keeble-Gagnère *et al.* 2018; Ramírez-González *et al.* 2018; The International Wheat Genome Sequencing Consortium (IWGSC) 2018) as well as pangenomic resources, which offer powerful insight for genomic architecture and structural variation (reviewed in Tiwari *et al.* 2024). However, genetic maps constructed from large populations remain useful for the investigation of nonmendelian inheritance and for highlighting regions of uncertainty in the sequence data (Deokar *et al.* 2014).

In this paper, we present an eight parent spring bread wheat MAGIC population. Three founders (Baxter, Westonia, and Yitpi) are Australian cultivars, which were previously used in a four-parent MAGIC population (Huang *et al.* 2012). The other five founders originated from Canada (AC Barrie), United States (Alsen), CIMMYT (Pastor), Israel (Volcani), and China (Xiaoyan54). All founders are spring wheats with the exception of Xiaoyan54 which is a winter wheat. Founders were chosen on the basis of genetic and phenotypic diversity, with a particular emphasis on diversity for wheat quality traits. All except Volcani have been grown commercially.

Within the single population presented here, we assess how increased recombination in three advanced levels of intercrossing increases mapping resolution. High-resolution genetic maps were constructed for lines at each level of intercrossing and compared with the physical map. Finally, we investigated evidence for localized segregation distortion due to large-scale introgression blocks.

## Methods

### Population

The founders for the population were three Australian cultivars (Baxter, Westonia, and Yitpi) and five founders representing global germplasm: AC Barrie (Canadian), Alsen (United States), Pastor (CIMMYT), Volcani (Israel), and Xiaoyan54 (China). Material was sourced from the Australian Winter Cereals Collection and CSIROs long-term germplasm store.

**Fig. 1.**
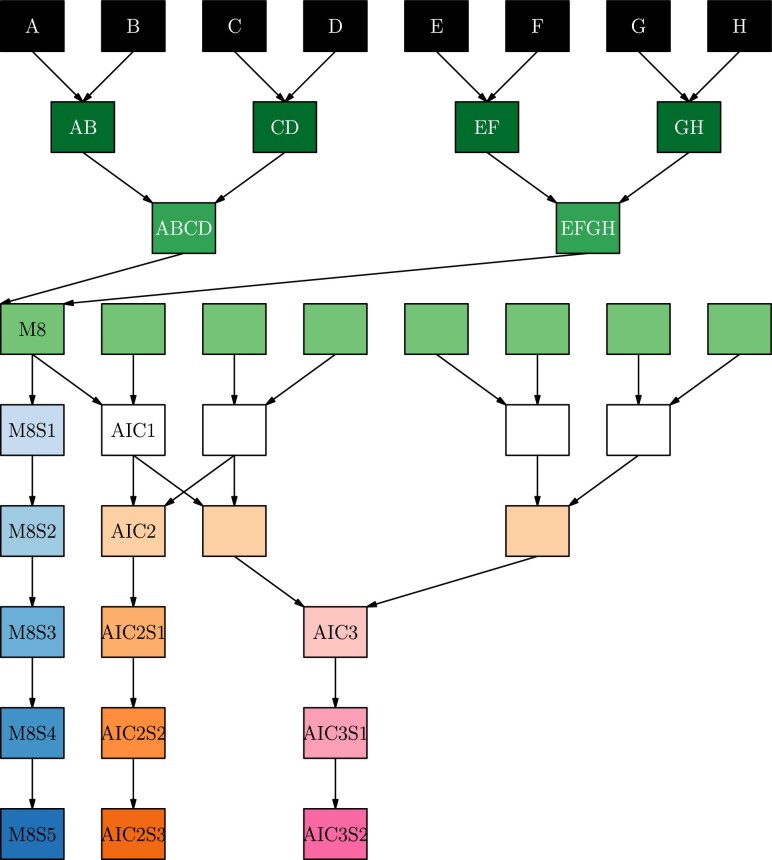
Schematic of the different branches of the three sub-populations. Color represents the phase of mixing/selfing; S* indicates generation of selfing. M8 lines undergo only mixing of the parents and inbreeding; AIC2 and AIC3 lines undergo two and three additional generations of intercrossing prior to inbreeding, respectively. This schematic represents a single funnel for each sub-population type, the progeny of which are subsequently referred to as MP8RIL, AIC2RIL, or AIC3RIL, see details in Methods.

**Fig. 2.**
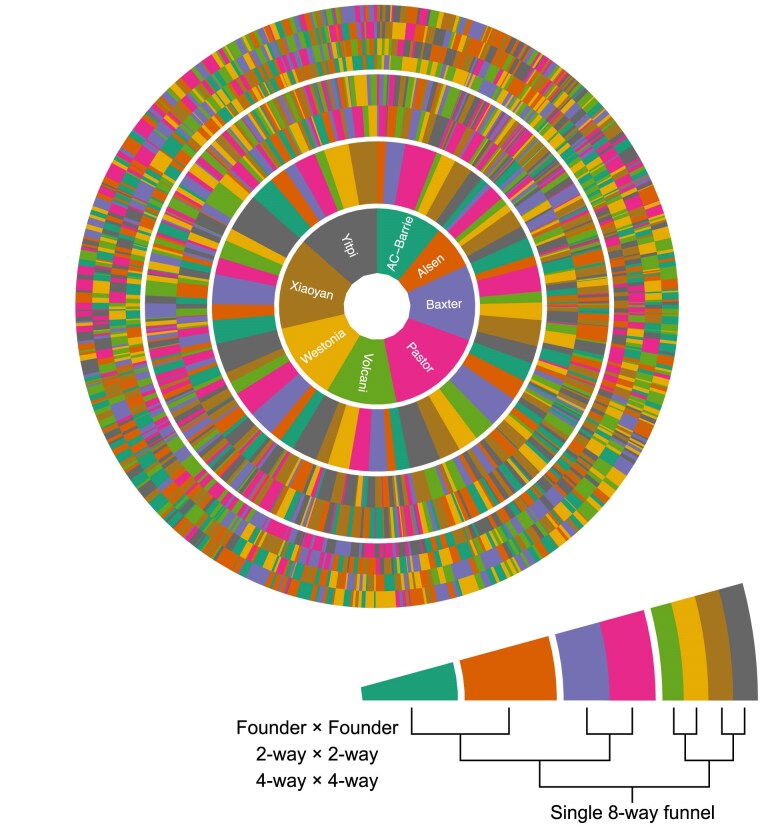
Radial representation of all 559 funnels used to generate the M8 RILs. An example single funnel is shown in the inset. Each ring in the figure represents a plant in each stage of the crossing strategy. The first, most central ring is the maternal founder in the first round of crossing. The second ring is the paternal founder in the first round. The third ring shows the founder makeup of the paternal 2-way line used in the second round of crossing. The outermost ring shows the founder makeup of the paternal 4-way line in the third round of crossing.

The population is divided into the three subpopulations shown in Fig. 1. For each subpopulation the pedigree has three stages, as described in Valdar *et al.* (2006). In the first stage, mixing, the parent genomes are combined together over three generations of mating. In the first generation, 28 crosses were made in a single direction (no reciprocal crosses). In the second generation, 210 crosses were made in a single direction. In the third generation, 589 distinct crosses were created, including reciprocal crosses in 276 cases. All unique combinations of founders, referred to as *funnels* are shown in Fig. 2, divided by each stage of the mixing process. The G3 individuals were common to all subpopulations and encompassed 313 of the 315 unique combinations possible for an 8-way population, excluding reciprocal crosses. Supplementary Fig. S1 shows the structure of the funnels, and the level of representation of each funnel at each stage in the final population. In the second stage, maintenance or intercrossing, individuals from different funnels were intercrossed to generate additional recombination between genome segments. There were 293, 286, and 745 lines in the first Advanced InterCross (AIC1), second (AIC2), and third (AIC3) generations of intercrossing, respectively. Finally, in the third stage, inbreeding, individuals were selfed for five generations. In all, there were 2,151 distinct crosses made to generate the complete population.

The first subpopulation (MP8RIL) contained 2,381 lines generated using mixing and selfing, but no generations of intercrossing. Of the possible 315 unique funnel combinations, 311 were used in the MP8RIL subpopulation. The second subpopulation (AIC2RIL) contained 286 lines generated using two generations of intercrossing prior to inbreeding. The third subpopulation (AIC3RIL) contained 745 lines generated using three generations of intercrossing prior to inbreeding.

### Genotyping and map construction

A preliminary map was constructed using five steps; genotype calling, recombination fraction estimation, linkage group identification, marker ordering, and map estimation. These steps were performed using R packages mpMap2, mpMapInteractive2, and magicCalling. Package mpMap2 (Shah and Huang 2019) is an R package for map construction using multiparent crosses, and is an updated version of mpMap (Huang and George 2011). Package mpMapInteractive2 (Shah 2019) is a package for interactively making manual changes to linkage groups and marker ordering, during the map construction process. Package magicCalling contains code used for SNP calling. Unless otherwise noted, all R functions are contained in mpMap2.

The preliminary map was iteratively improved until a final estimated map was constructed. For example, some marker calling errors cannot be accurately identified without a genetic map; see Supplementary Figs. S2a–S2c for an example.

#### Genotype calling

Each marker was first processed using the data normalization approach of GenCall (Peiffer *et al.* 2006), and then converted to the polar coordinates (r,θ). Markers were called using either Density-Based Spatial Clustering of Applications with Noise (DBSCAN) (Ester *et al.* 1996) or the Hierarchical Bayesian Clustering (HBC) model described in Shah and Whan (2018). The HBC model is implemented using the JAGS library (Plummer 2015). It has the advantage of calling heterozygotes, but the disadvantage that it cannot be applied if there are more than two marker alleles. This can occur if there are secondary polymorphisms at the target location, or if a marker is polymorphic at multiple locations on the genome. We chose parameters for the HBC method that resulted in fairly aggressive calling of heterozygotes.

DBSCAN can identify more than two marker alleles for a single marker. However, it cannot identify heterozygotes, and has two parameters (minPts and ε) that must be specified manually for each marker.

These methods have advantages over the GenCall algorithm implemented in Illumina GenomeStudio; DBSCAN can call more than two marker alleles, while the HBC approach can call heterozygotes.

We performed genotype calling by first applying HBC to every marker. We identified monomorphic markers, and lines that had a high error rate. These lines were removed, and HBC was reapplied to the polymorphic markers. A subset of the fitted HBC models were reviewed manually, based on some simple heuristics. Reasons for needing to review a marker included nonconvergence of the Markov Chain Monte Carlo (MCMC) algorithm used to fit the HBC model, an unreasonably high rate (>0.06) of heterozygote calls, or the presence of more than two marker alleles. If more than two marker alleles were found, DBSCAN was used to define clusters. Both DBSCAN and the HBC model are implemented in the magicCalling package.

#### Estimation of recombination fractions

Recombination fractions between all 437,059,395 pairs of called markers were estimated using the function estimateRF. Estimation was performed using numerical maximum likelihood, with 61 possible recombination fraction values. This step took 14 h, and required 300 GB of memory. Chromosome 2B carries the Sr36 introgression from *Triticum timopheevi* (Tsilo *et al.* 2008), which is known to distort genetic inheritance. This was corrected for in later steps, using the weighting method described in Shah *et al.* (2014). The weight assigned to a particular line depended not only on whether the introgression was present or absent, but the number of generations of intercrossing.

#### Construction of linkage groups

The set of all markers was divided into 400 smaller groups, by applying hierarchical clustering to the matrix of recombination fractions, using the function formGroups. The underlying implementation comes from the fastcluster package (Müllner 2013). These small groups were then aggregated by hand using mpMapInteractive2, resulting in 29 groups of appreciable size (¿10 markers). The linkage groups corresponding to the 21 chromosomes were identified based on the consensus map (Wang *et al.* 2014). In some cases, it was difficult to identify the correct linkage group for a chromosome, especially for the D chromsomes.

#### Marker ordering

Ordering of chromosomes proceeded in three steps. The first step was performed using the clusterOrderCross function. Each chromosome was divided into 30 subgroups using hierarchical clustering. These subgroups were used to define a 30×30 matrix, where each entry was the average of the recombination fractions between markers in those subgroups. The 30 subgroups were automatically ordered by applying antirobinson serialization (Brusco *et al.* 2007; Hahsler *et al.* 2008) to this 30×30 matrix. At the end of this step, the ordering of markers *within* a subgroup is still arbitrary. An example of this step is shown in Supplementary Figs. S3a–S3c.

The second step was the ordering of markers within chromosomes, using the orderCross function. This function applies a modified version of antirobinson serialization to the matrix of recombination fractions. Standard antirobinson serialization allows global changes to the marker ordering. The modified version only makes local changes to the ordering, and is therefore computationally much faster. The result of this step is shown in Supplementary Fig. S3d.

The third step was manual changes made using the mpMapInteractive2 package, based on visual inspection of the recombination fraction heatmaps.

#### Map distance estimation

Map distance estimation was performed by forming a collection of equations, and approximately solving them using nonlinear least squares. These equations allow the genetic distances between pairs of markers, which are not adjacent in the chosen ordering, to be used as part of the estimation process. Consider the case where there are three markers, known to be in the correct order. If the matrix of estimated genetic distances (obtained from the estimated recombination fractions) is


$$\left(\begin{array}{ccc} 0 & 5 & 15\\ 5 & 0 & 7\\ 15 & 7 & 0 \end{array}\right),$$

then the matrix equation to be solved is


$$\left(\begin{array}{cc} 1 & 0\\ 1 & 1\\ 0 & 1 \end{array}\right)\left(\begin{array}{c} a_{1}\\ a_{2} \end{array}\right)=\left(\begin{array}{c} 5\\ 15\\ 7 \end{array}\right),$$

where $a_{1}$ is the distance between the first and second markers, and $a_{2}$ is the distance between the second and third markers. This method of estimating the genetic map is implemented by the estimateMap function. See Shah and Huang 2019 for further details.

#### Incremental changes

After an initial map was constructed, it was improved incrementally, resulting in the map presented in this paper. Incremental changes are necessary, as some improvements are only possible *after* a preliminary map has been produced. Three types of incremental changes were made. The first was the deletion of markers that were found to be badly called (based on visual inspection of the HBC models), or polymorphic on multiple chromosomes. The second was the addition of markers that could be localized to specific genetic regions, using the preliminary genetic map and a QTL mapping approach, with marker data being used as a trait.

The third was the identification of dangling linkage groups, as being part of a specific chromosome. In particularly marginal cases, we relied on the International Wheat Genome Sequencing Consortium (IWGSC) RefSeq v1.0 sequence.

### Recombination

Recombination events were imputed by assuming a Hidden Markov Model (HMM) for the identity-by-descent genotypes. This assumption is not exact, but is known to be highly accurate as long as the distances between consecutive markers are not large. The HMM assumption allows the application of the Viterbi algorithm to impute the most likely underlying genotype, for each line, including imputation of heterozygosity. The Viterbi algorithm is implemented in mpMap2. We used an error parameter of 0.1. The underlying probability computations build on previous published work (Teuscher and Broman 2007; Broman 2012).

### Segregation distortion

We ignored marker heterozygotes throughout our assessment of segregation distortion.

#### Main effects

We performed tests for segregation distortion at a locus as follows. For each potential genotype at that position, we computed the average, over all genetic lines, of the probability that this genotypes occurred in each line. These values are then arranged into a vector, and the standard chi-squared test for independence was performed.

This statistic is generated from a “contingency table” of noninteger values, however it is still a valid test (central limit theorem). We applied this test for all genetic locations, using a 1 cM grid of equally spaced points.

#### Pairwise effects

Genomic incompatibilities between parents can be detected by further analysis of segregation distortion on the level of interactions. We performed tests for the presence of interactions between a pair of genetic locations as follows. For each potential combined genotype at these positions, we computed the average, over all genetic lines, of the probability that these genotypes occurred in each line. These values are then arranged into a 8×8 matrix, and the standard chi-squared test for independence was performed. An example of the matrix used in this test is shown in Supplementary Table S1. The top-left value is the average over all genetic lines of $P(L_{1}=\text{AC-Barrie},L_{2}=\text{AC-Barrie})$, where $L_{1}$ and $L_{2}$ are genetic locations. In this case, the value is around 0.01.

This statistic is generated from a “contingency table” of noninteger values, however it is still a valid test. We applied this test for all pairs of genetic locations on different chromosomes, using a 1 cM grid of equally spaced points.

#### Sex-specific effects

We also investigated segregation distortion that occurs in specific cross combinations (funnels). For each line in the MP8RIL subpopulation, one founder was maintained as a maternal contribution in every cross, and one was maintained as a paternal contribution in every cross. For each founder *f*, we took two subsets of the MP8RIL population—those which had *f* as only a maternal contribution, and those which had *f* as only a paternal contribution. For each genetic location on the 1 cM grid considered previously, we performed a chi-squared test for the presence of a sex-specific effect on the inheritance of *f*. Similar to the interactions, these tests were based on sums of haplotype probabilities.

#### Funnel effects

Another cause of segregation distortion is potential incompatibilities between pairs of founders in the initial cross, which may lead to segregation distortion in the final generation. For each founder *f*, we separated the MP8RIL lines into seven groups, based on the founder which was crossed with *f* in the first generation. We ignored the sex of *f* when forming these groups. We then performed a chi-squared test for differences in genetic inheritance of *f* between these seven groups. This tests the presence of a funnel-specific effect, for the inheritance of founder *f*. Similar to the interactions, these tests were based on sums of haplotype probabilities.

## Results and discussion

### Genotype calling

Genotyping was performed for lines from all subpopulations using the Infinium iSelect 90 K (Wang *et al.* 2014) SNP assay. This resulted in data for 81,587 markers. At the end of the marker calling process there were 29,566 polymorphic markers, of which 6,743 were reviewed manually. Almost all markers were called with the HBC method while most markers called with the DBSCAN method had either two or three alleles Supplementary Fig. S4.

Almost all markers had a heterozygote frequency <0.05 and a median of 0.025 Supplementary Fig. S5. This is slightly more than would be expected for five generations of selfing with (0.0273), and without additional intercrossing (0.0312). Note that *marker heterozygotes* are not the same as identity-by-descent heterozygotes; if two parents carry the same marker allele, then a heterozygote for those parents will not be a marker heterozygote. So the proportion of marker heterozygotes is lower than the proportion of identity-by-descent heterozygotes, and Supplementary Fig. S5 shows a situation where the proportion of marker heterozygotes is generally higher than expected. This is partially due to our fairly aggressive calling of marker heterozygotes or could also be due to the presence of homologs. A complete assessment of heterozygosity using identity-by-descent heterozygotes using all markers *jointly* may be conducted in future analyses.

Calling for most markers was straightforward, however there were a small number of challenging cases that were initially misscalled. These cases are only identifiable *after* a genetic map has been constructed. For example, marker Kukri_c37840_253 is present on chromosomes 2A and 2B, and polymorphic on both, although with a genetic map this is not obvious (Supplementary Fig. S2a). In Supplementary Fig. S2b, color represents the imputed genotype at a location on chromosome 2B. In Supplementary Fig. S2c, color represents the imputed genotype at a location on chromosome 2A. As this pattern cannot reasonably have arisen by chance, it is clear that there are four clusters here. Without a genetic map there will appear to be only two clusters, and calling of this marker will be incorrect. The marker will appear to map to two chromosomes, although due to the incorrect calling it will not be correct on either chromosome.

Supplementary Table S2 summarizes the number of markers with segregation distortion, on each chromosome. However, this potentially says more about the markers than the underlying genetic structure; the ability to successfully call marker alleles varies across markers, and across marker alleles for each marker. So observed segregation distortion at the individual marker level is susceptible to effects relating to the marker calling algorithm, and the ease with which different marker alleles can be called.

### Map summary

Our constructed map has 27,687 markers across all 21 chromosomes. Supplementary Table S2 provides a complete summary of the mapped markers. Markers that are distorted are still present in the map. Removing markers on the basis of single-locus distortion is difficult, as the distortion may be due only to a difference in the marker calling rate for different marker alleles.

Supplementary Fig. S6 shows the positions of all markers, and all gaps in the map, with color representing the number of unique positions per centiMorgan. Supplementary Fig. S7 is similar, but color represents the number of markers per centiMorgan. Chromosome 2B has been estimated as being very long; this is an unavoidable result of the large number of markers on that chromosome. As marker density increases, distances between adjacent markers become extremely small. As a result, any estimation error will almost certainly be in the direction of estimating a distance that is too large. Compounding the problem, there are more such estimates to be made. Genotyping error may also lead to the separation of markers that do not have any true recombination event separating them. We note that the relevant inputs to QTL mapping algorithms, such as genotype probabilities and imputed genotypes, are relatively insensitive to overall chromosome length.

The marker densities of 1.58 unique positions/cM on the A genome and 2.02 unique positions/cM on the B genome were far higher than the marker density of 0.50 unique positions/cM for the D genome. Around 37%, 55%, and 6% of markers were mapped to the A, B, and D genomes respectively. The lower coverage on the D chromosomes is reflected both in the length (parts missing) and the resolution (bigger gaps). This feature is known to reflect the lower polymorphism of the D genome (Wang *et al.* 2014). However, it *may* also be related to the presence of markers polymorphic on multiple chromosomes. For example, we have noted a large number of markers polymorphic on both 2B and 2D. These markers are not simple to map, and have not been included. If a large fraction of markers on the D genomes are also present on the A or B genomes, and cannot be mapped, this could be interpreted as reduced polymorphism on the D genome.

In general, the number of unique positions per chromosome was far lower than the number of markers mapped. The 27,687 markers were mapped to 7,674 unique positions.

### Map comparison and validation

The map constructed in this population has high coverage, density and resolution. While the 90 K consensus map (Wang *et al.* 2014) contains nearly 47,000 markers, the eight individual biparental populations contributing to this map contains fewer than 19,000 markers each. Another previously published consensus map (Li *et al.* 2015) has 28,000 markers at 3,757 unique positions, but for each of the three populations merged to form the consensus, fewer than 20,000 markers were mapped. Genotyping by sequencing can detect a very high number of markers, resulting in genetic maps with extremely high marker density, but without increasing map resolution. For example, 1.7 million markers in 1,335 bins were reported in a doubled haploid population of 90 lines, demonstrating that the number of unique locations is limited by the recombination observed in the population (Chapman *et al.* 2015). More recently in a 16 parent wheat MAGIC recombined over four levels of intercrossing, exome capture sequencing revealed 1.2 million SNP and a pruned subset 55 K markers were mapped to 23,000 unique genetic map positions (Scott *et al.* 2021; Fradgley *et al.* 2023) and a previously reported map developed with 643 lines from an 8-way wheat MAGIC population (referred to subsequently as NIAB 8-way) has 18,601 markers in 4,578 unique positions (Gardner *et al.* 2016).

To validate the ordering of the markers in the map we report here, we compared 90 K SNP genetic map positions with the 90 K consensus map (Wang *et al.* 2014) and with the NIAB 8-way map (Gardner *et al.* 2016). There was overall agreement between the maps (Supplementary Figs. S8 and S9, with the exception of a region with a large number of conflicts on chromosome 2B due to the Sr36 introgression (Tsilo *et al.* 2008). The introgression is rarely broken up by recombination events (see discussion of recombination) and displays distorted inheritance (see discussion of segregation distortion main effects). This makes map construction for chromosome 2B difficult using this population. A summary of markers with agreeing and conflicting positions is shown in Supplementary Table S3.

Supplementary Fig. S10 compares our map and the IWGSC RefSeq v1.0. Large disagreements between the physical map and the MAGIC map may indicate differences in genome ordering between Chinese Spring and the parents used for mapping. They may also be due to paralogous sequences, or genetic insertions, deletions, and inversions as has been recently found through the multiple wheat reference genomes (Walkowiak *et al.* 2020). On a fine scale, the order of markers in the genetic map and the IWGSC RefSeq v1.0 are not expected to match exactly, due to variability in recombination fraction estimates affecting the ordering in the MAGIC map. We do not expect to have high enough resolution in the MAGIC map to match the resolution of the sequence data; it has previously been demonstrated that a genetic map is insufficient to completely order scaffolds in tomato (Shearer *et al.* 2014). However, the high resolution of the MAGIC map can be used to highlight regions of uncertainty or dissonance in the physical map and improve reference assemblies (Deokar *et al.* 2014).

### Recombination

Comparison of the three subpopulations allows assessment of the benefit of the additional intercrossing, since AIC2RIL and AIC3RIL have two and three extra rounds of crossing, respectively. Supplementary Table S4 shows the average number of recombination events for each chromosome, for each of the three subpopulations. Additional generations of intercrossing lead to a noticeable increase in recombination events. The imputed number of recombination events for chromosome 3B was much higher than the value of 2.6 in (Choulet *et al.* 2014), highlighting the high level of recombination in the MAGIC population. Supplementary Fig. S11 shows the distribution of the number of recombination events for all three populations.

The imputed haplotype blocks were used to estimate the average haplotype block size, at different points on the genome, for all three subpopulations. Block sizes clearly decreased towards the ends of each chromosome and additional levels of intercrossing in the three subpopulations successfully reduced block sizes even at the chromosome centres Supplementary Fig. S12. Both chromosomes 2B and 6B that contain introgressions that distort genetic inheritance as described in Section 3, had large average haplotype block sizes.

The haplotype blocks on chromosome 2B are particularly interesting. Supplementary Fig. S13 shows the average sizes of the haplotype blocks, classified according to their underlying genotype. The influence of the Sr36 introgression is obvious in the much larger size of the Baxter haplotype blocks. Supplementary Fig. S13 also shows an interesting spike in the average size of the Volcani haplotype blocks around 400 cM. This is difficult to interpret, as the difference seems to be due to a *decrease* in the number of small Volcani haplotype blocks at around 400 cM.

### Segregation distortion

#### Main effects

Individual markers displaying distortion of segregation from that expected under Mendelian assumptions may indicate genotyping error. However, large groups of distorted markers may indicate biologically relevant phenomena, such as introgressions or translocations of genetic material. We have previously demonstrated that such regions can be mapped successfully in MAGIC populations (Huang *et al.* 2012; Shah *et al.* 2014).

**Fig. 3.**
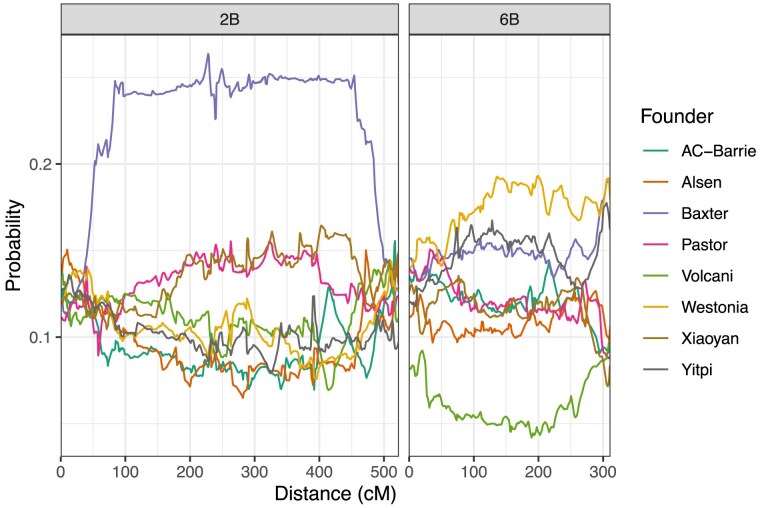
Average genetic composition across the entire population, for chromosomes 2B (left) and 6B (right).

Supplementary Fig. S14 gives a plot of the chi-squared statistics, testing for segregation distortion at the identity-by-descent level. The contribution of each founder across the full genome is shown in Supplementary Fig. S15. We note (Supplementary Table S2, Fig. 3) a large region of segregation distortion on Chr 2B. At the most distorted point on chromosome 2B, Baxter is inherited by 26% of the final population, instead of the expected 12.5%. At the most distorted point, the chi-squared test statistic for this effect is 705, and the associated *P*-value is numerically equal to 0. In light of this *P*-value, and the presence of distortion across a large region of chromosome 2B, it is clear that this is real genetic effect. This distortion identifies an introgression known as Sr36 (Tsilo *et al.* 2008), which is contributed by the parent Baxter and is known to undergo meiotic drive.

Based on cytogenetic analysis of Baxter (C. Cavanagh, personal communications) the long arm of Chromosome 2B is replaced with 2G from *Triticum timopheevi*. According to (Badaeva *et al.* 1996), Chromosome 2G is substituted for 2B at a frequency higher than expected, and suggested and may carry putative homoeoalleles of gametocidal genes present on group-2 chromosomes of several alien species. Chromosome 2G is recovered at a higher than expected frequency in the progeny of a hexaploid derivative of a cross between wheat and T. araraticum that was heterozygous for chromosomes 2G and 2B (Brown-Guedira *et al.* 1996).

The markers on chromosome 2B with the highest distortion are located between 299 cM and 322 cM in our map. This region was identified by first manually choosing ten markers known to be part of the introgression. These markers were all specific for the Baxter allele, and highly distorted. We then identified other markers specific for the Baxter allele, which were extremely strongly linked to the ten which were manually selected. As shown in Supplementary Figs. S6 and S7, this region contains a large number of markers.

The proportion of lines containing the introgression appears to depend on the number of generations of intercrossing (Table 1). It is clear that lines with intercrossing carry the introgression more frequently than those without intercrossing. It would also be expected that AIC3RIL lines should carry the introgression more frequently than AIC2RIL lines, but this is not observed; this may be due to the smaller sample size for the AIC2RIL and AIC3RIL subpopulations, compared with the MP8RIL subpopulation.

**Table 1.** Genetic composition at position 311 cM on chromosome 2B, which has the highest rate of Baxter alleles on chromosome 2B.

Subpopulation	AC-Barrie	Alsen	Baxter	Pastor	Volcani	Westonia	Xiaoyan	Yitpi
All	0.08	0.09	0.26	0.14	0.11	0.09	0.14	0.09
MP8RIL	0.09	0.09	0.23	0.13	0.12	0.10	0.14	0.10
AIC2RIL	0.07	0.08	0.30	0.16	0.07	0.09	0.14	0.10
AIC3RIL	0.07	0.07	0.27	0.16	0.08	0.10	0.16	0.07

The presence of the introgression makes estimated recombination fractions somewhat unreliable, despite our use of a correction. As the estimation of map distances is based on estimated recombination fractions, this has the effect of inflating the length of chromosome 2B. Without a reliable model of genetic inheritance, there is little that can be done to fix this, short of rescaling the positions of all markers on that chromosome. The high marker density also contributes to the inflated length of this chromosome.

On chromosome 6B, Volcani is under-represented across most of the chromosome, being present in 4.2% of the final lines at position 190 cM, which is the point on 6B with the lowest rate of Volcani alleles. Westonia alleles are also inherited more frequently than expected, at this point. Table 2 shows the genetic composition of the population at 190 cM on chromosome 6B, for the entire population and all three subpopulations. The proportion of Volcani alleles decreases as the number of generations of intercrossing increases, with only 2% of AIC3RIL lines carrying the Volcani allele. This suggests that the Volcani allele is inherited <50% of the time in every generation.

**Table 2.** Genetic composition at position 190 cM on chromosome 6B, which has the lowest rate of Volcani alleles on chromosome 6B.

Subpopulation	AC-Barrie	Alsen	Baxter	Pastor	Volcani	Westonia	Xiaoyan	Yitpi
All	0.13	0.11	0.15	0.11	0.04	0.18	0.12	0.15
MP8RIL	0.12	0.12	0.15	0.11	0.05	0.19	0.12	0.15
AIC2RIL	0.13	0.14	0.15	0.12	0.03	0.20	0.09	0.13
AIC3RIL	0.15	0.09	0.16	0.14	0.02	0.17	0.11	0.17

The chi-squared test statistic for distortion at 190 cM on chromosome 6B is 325, and the associated *P*-value is numerically equal to 0. In light of this *P*-value, and the presence of distortion across a large region of chromosome 6B, it is clear that this effect is statistically significant. As the distortion occurs almost chromosome-wide, it is not possible to determine the position of any genetic cause.

There is a further distortion on 6B, specific to the end of the chromosome. We estimate the position of this distortion as 308 cM. At this position, the Baxter, Westonia and Yitpi alleles are all inherited more frequently than expected. Table 3 gives the genetic composition at this point, for the entire population and all three subpopulations. Interestingly, additional generations of intercrossing seem to increase the proportion of Westonia alleles. One potential cause for segregation distortion on Chromosome 6B could be the introgression from wild emmer wheat (*Triticum turgidum* ssp. *dicoccoides*) present in the Volcani founder which carries the NAM-B1 gene, also known as GPC-B1 (Uauy *et al.* 2006; Distelfeld *et al.* 2012).

**Table 3.** Genetic composition at position 308 cM on chromosome 6B, which has the highest rate of Baxter alleles on chromosome 6B.

Subpopulation	AC-Barrie	Alsen	Baxter	Pastor	Volcani	Westonia	Xiaoyan	Yitpi
All	0.09	0.10	0.19	0.09	0.09	0.19	0.07	0.17
MP8RIL	0.10	0.10	0.19	0.09	0.10	0.18	0.08	0.17
AIC2RIL	0.08	0.10	0.18	0.09	0.07	0.20	0.10	0.18
AIC3RIL	0.08	0.11	0.19	0.07	0.09	0.23	0.05	0.18

Other localized regions of segregation distortion occur on chromosomes 2D, 4A, and 7D. The most distorted point on chromosome 2D occurs at 155 cM; this is likely due to a genetic interaction (discussed in the next section). Supplementary Table S5 gives the genetic composition at 155 cM. The most distorted point on chromosome 4A occurs at 136 cM; Supplementary Table S6 gives the genetic composition at this point. The most distorted point on chromosome 7D occurs at 72 cM; Supplementary Table S7 gives the genetic composition at this point. The effect on chromosome 7D may be due to a funnel effect; see the discussion of segregation due to funnel effects. There is another region of distortion at the end of chromosome 7D, at 238 cM (Supplementary Table S8). The genetic composition at this second position on chromosome 7D is substantially different to the composition at the first distorted position. There is also evidence for distortion on chromosomes 1D and 5A.

The *P*-values for a test for distortion are numerically equal to 0 for the localized distortions on chromosomes 2D, 4A and 7D. For localized distortion, *P*-values should be interpreted extremely cautiously. They may, for example, indicate mapping or genotyping errors, which invalidate the underlying assumptions (Greenland *et al.* 2016). Our use a genotyping error rate parameter in haplotype probability computation and imputation tends to guard against these types of errors.

#### Pairwise effects

Interactions were identified as “significant” if the *P*-value was smaller than $10^{-9.5}$; this fairly strict threshold was chosen to account for multiple hypothesis testing. There are two significant interactions.

The first is between position 445 cM on chromosome 2B and position 127 cM on chromosome 2D. Although we give the most significant position, the range of locations for which this interaction is significant is very large, especially on chromosome 2B. A Table showing the most significant 2B–2D interaction is given in Supplementary Table S1. A visual representation of the interaction is shown in Supplementary Fig. S16, where color represents the observed frequency as a multiple of the expected frequency under independence. Dark blue represents combinations present much more frequently than expected under independence. We see that lines with Baxter alleles at both locations occur much more frequently than expected. We also see that lines with a Baxter allele on chromosome 2B and a Xiaoyan allele on chromosome 2D occur much less frequently than expected. We note that the segregation distortion detected at 155 cM on chromosome 2D may be caused by the interaction term detected here.

This interaction might be due to an issue with marker calling for markers on chromosomes 2B and 2D. The large segment of *timopheevi* introgrogression (most of the long arm of 2B) means that the *aestivum* 2B chromosome is missing. So the “normal” hybridization state for many 2D markers will appear to have a lower copy number and potentially a theta shift. The interaction might also be due to an issue with the map construction process, with some markers being mapped to the wrong chromosome. However, an extensive search for mapping errors failed to identify problems that could be responsible for this interaction.

The second significant interaction is between chromosomes 2B and 6B, and occurs over a more limited region. The location on chromosome 6B (306 cM) is almost the same as one of the locations identified for a main effect, so a genetic interaction may be the cause of that main effect. Supplementary Table S9 shows the joint distribution of the underlying alleles at position 306 cM on chromosome 6B and position 462 cM on chromosome 2B. A visual representation of the interaction is shown in Supplementary Fig. S17.

The nominal significance threshold of $10^{-9.5}$ is extremely conservative, and there are likely to be other interaction terms. This conversatism is necessary because, in our experience, markers polymorphic on multiple chromosomes can introduce an erroneous (yet highly significant) interaction. Using the Bonferroni correction to account for the 22,992,937 tests results in an adjusted significance threshold of 0.0073. The Bonferroni correction is likely to be extremely conservative in this case. Use of *P*-values assumes correctness of the genetic map and the marker calling process; *P*-values should be used with caution.

#### Sex-specific effects

A plot of the chi-squared test statistics for the presence of a sex-specific effect is shown in Supplementary Fig. S18, for every founder and every position. There is one very clear effect on chromosome 6B, related to the sex of the Westonia line in the initial cross. The most significant position for this effect is 158 cM. At this point, the Westonia allele is present in 19% of lines. For lines where Westonia is always a maternal contribution, the Westonia allele is present in 9% of lines. For lines where Westonia is always a paternal contribution, the Westonia allele is present in 29% of lines. The nominal *P*-value of this effect (ignoring multiple testing) is $8.32\times 10^{-10}$.

#### Funnel effects

Next, we consider effects relating to the specific combinations of founders in the original cross (funnels). A plot of the chi-squared test statistics for every founder is shown in Supplementary Fig. S19. Table 4 shows effects at three loci with major effects. There is an obvious effect relating to founder Volcani on chromosome 6B, which appears to occur chromosome-wide; however, it statistically greatest at 0 cM. If founder Volcani is crossed with Westonia, Xiaoyan or Yitpi in the first generation, then the haplotype on 6B is extremely unlikely to contain any Volcani alleles. If Volcani is crossed with Baxter in the initial cross, then inheritance of Volcani alleles on 6B is inflated. Table 4 understates the size of the effect, particularly with respect to Westonia/Volcani crosses. Out of 355 MP8RIL lines that cross Westonia and Volcani in the first generation, only 1 has >0.2 probability of having a Volcani allele at 0 cM. It is possible that no lines from these crosses carry a Volcani allele at this position.

**Table 4.** Proportion of alleles contributed by Volcani or Pastor (denoted in Founder column) at three loci of interest.

Chromosome	Distance (cM)	Founder	ACBarrie	Alsen	Baxter	Pastor	Volcani	Westonia	Xiaoyan	Yitpi
6B	0	Volcani	0.12	0.15	0.18	0.10		0.01	0.02	0.03
3B	299	Pastor	0.11	0.15	0.01		0.13	0.10	0.11	0.01
7D	12	Pastor	0.13	0.13	0.01		0.09	0.12	0.11	0.01

There are blank values in the Volcani and Pastor columns where a selfing would occur.

As the Volcani funnel effect on chromosome 6B occurs chromosome-wide, it also occurs at the location on chromosome 6B where we previously detected segregation distortion (308 cM), and at the location where we previously detected an interaction term (306 cM). It is possible all three of these effects have the same underlying genetic cause.

There are two slightly less significant effects relating to Pastor. The first is at 299 cM on chromosome 3B, with a nominal *P*-value of $3.1\times 10^{-13}$. If Pastor is crossed with Baxter or Yitpi in the first generation, then it is unlikely to find a Pastor allele at 299 cM on chromosome 3B. Out of 341 MP8RIL lines that cross Pastor and Yitpi in the first generation, only 5 lines have >0.5 probability of having a Pastor allele at 299 cM. Out of 332 MP8RIL lines that cross Pastor and Baxter in the first generation, only 2 lines have >0.5 probability of having a Pastor allele at 299 cM.

There is another effect relating to Pastor on chromosome 7D at position 12 cM, with a nominal *P*-value of $8.0\times 10^{-12}$. If Pastor is crossed with Baxter or Yitpi in the first generation, then it is unlikely to find a Pastor allele at 12 cM on chromosome 7D. Out of 341 MP8RIL lines that cross Pastor and Yitpi in the first generation, only 4 lines have >0.5 probability of having a Pastor allele at 12 cM. Out of 332 MP8RIL lines that cross Pastor and Baxter in the first generation, only 3 lines have >0.5 probability of having a Pastor allele at 12 cM.

It is highly unlikely for a funnel effect to arise by chance; most types of errors (e.g. mapping errors, genotyping errors) would not cause such an effect. So the effects on chromosomes 3B and 7D represent *at least one* funnel effect. But the funnel effects on chromosomes 3B and 7D appear very similar; it is conceivable that there is only a *single* effect which, due to a mapping error, (wrongly) appears in two different regions. The most significant interaction between positions 295–303 cM on chromosome 3B and positions 8–16 cM on chromosome 7D has a *P*-value of 0.24. This suggests that there may be two separate effects.

### Heterozygosity

Supplementary Fig. S20 shows the distribution of the proportion of residual heterozygosity, as determined by the Viterbi algorithm. There are 75 lines with a proportion or residual heterozygosity >0.2. This is likely due to mistakes during the selfing stages of the pedigree, where a line was outcrossed instead of being self-pollinated; it is difficult to guarantee that selfing occurs.

The proportion of residual heterozygosity identified by the Viterbi algorithm was 0.0146 for the MP8RIL lines, 0.034 for the AIC2RIL lines and 0.0273 for the AIC3RIL lines. The theoretical expected residual heterozygosity proportions are 0.0312 without intercrossing, and 0.0273 with intercrossing. While the values for the AIC2RIL and AIC3RIL populations roughly agree with the theoretically expected proportion, the MP8RIL subpopulation contains substantially less residual heterozygosity than expected.

### Conclusion

We have presented a large, densely genotyped, high-resolution mapping population and demonstrated its use for a variety of investigations providing new insight into genomic structure in wheat. We have validated a high-density map with the highest resolution currently available in any single wheat population, and used it to identify recombination breakpoints and characterize regions of segregation distortion. Notably, on chromosome 6B we found evidence for segregation distortion, an interaction term, a sex-specific effect *and* a funnel effect. It is unclear how many underlying genetic causes there are for these effects. Identification of these types of effects is crucial for translational research in crops, as they lead to a deeper understanding of genomic structure and its influence on breeding decisions. This population provides a high-value genetic resource useful for better understanding basic wheat genetics and improving the crop.

Here we have focused on analysis of genomic structure facilitated by the large number of lines genotyped at high density. This population has undergone phenotyping for a large number of traits, which almost always display transgressive segregation. In future studies, we hope to gain some insight into the effect of segregation distorting genetic effects on phenotypic performance.

By building on the foundation presented here with further investigation of how the genomic structure contributes to phenotypic diversity, we expect that our understanding of the importance of structural diversity in wheat can be better understood and utilized for wheat improvement.

## Supplementary Material

jkaf037_Supplementary_Data

## Data Availability

The genetic map, along with the genetic data for the population, and imputed genotypes, are available online (Shah *et al.* 2018). This analysis was performed using packages mpMap2 (Shah and Huang 2019), mpMapInteractive2, and magicCalling (Shah and Whan 2018), which are all publicly available (Shah 2018, 2019) from Github as cited. Due to age and viability, seed material is unavailable. Supplemental material available at G3 online.
