# Supplementary material for: Recombination and structural variation in a large 8-founder wheat MAGIC population
Source: G3 (Bethesda). 2025 Feb 21;15(4):jkaf037. doi: 10.1093/g3journal/jkaf037 (PMC12005149; doi:10.1093/g3journal/jkaf037)
Supplement: jkaf037_Supplementary_Data [file jkaf037_supplementary_data.pdf]

	AC-Barrie	Alsen	Baxter	Pastor	Volcani	Westonia	Xiaoyan	Yitpi	Total
AC-Barrie	0.01	0.01	0.00	0.01	0.01	0.01	0.03	0.01	0.10
Alsen	0.01	0.01	0.00	0.01	0.01	0.01	0.02	0.01	0.08
Baxter	0.03	0.03	0.07	0.03	0.02	0.02	0.01	0.03	0.25
Pastor	0.01	0.01	0.00	0.01	0.01	0.02	0.03	0.01	0.12
Volcani	0.01	0.02	0.00	0.02	0.01	0.01	0.02	0.01	0.10
Westonia	0.01	0.01	0.00	0.01	0.01	0.01	0.02	0.01	0.09
Xiaoyan	0.02	0.02	0.00	0.02	0.02	0.02	0.04	0.02	0.16
Yitpi	0.01	0.01	0.00	0.01	0.01	0.01	0.03	0.01	0.10
Total	0.12	0.13	0.08	0.13	0.11	0.11	0.21	0.12	

Table S1: Observed distribution of alleles, for the most significant 2B-2D interaction. Rows represent the genotype at position 445 cM on chromosome 2B. Columns represent the genotype at position 127 cM on chromosome 2D.

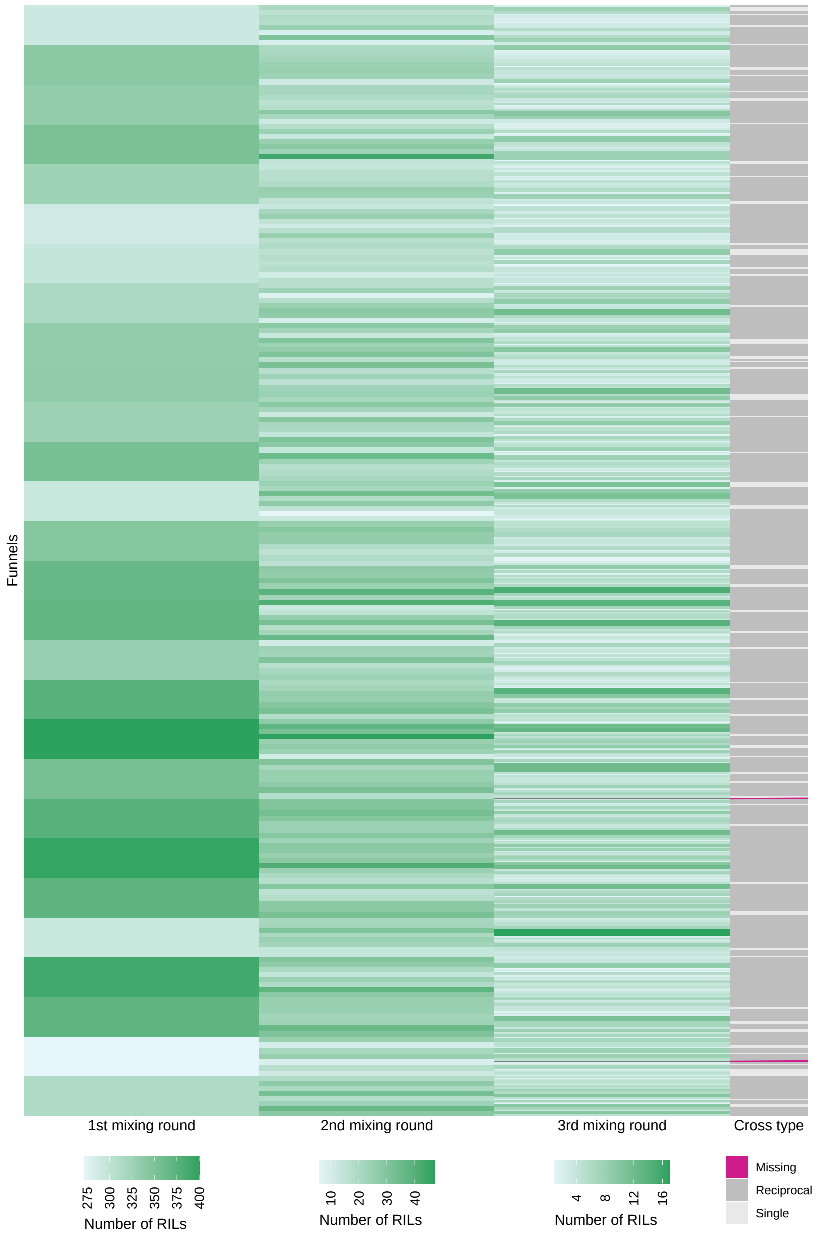

Figure S1: Heatmap representation of funnels in final population, according to the maternal parent in each round. Each rectangle represents a single cross. The rectangles are coloured according to how many MP8RILs were derived from that cross in the final population. The rectangles are arranged in funnels so that crosses in each round of mixing are positioned to the right of the maternal cross from the previous round. The two missing crosses in the third round of mixing are highlighted in red. At the far right, crosses in the third round that were performed in both directions (reciprocal crosses), are shown by a grey line.

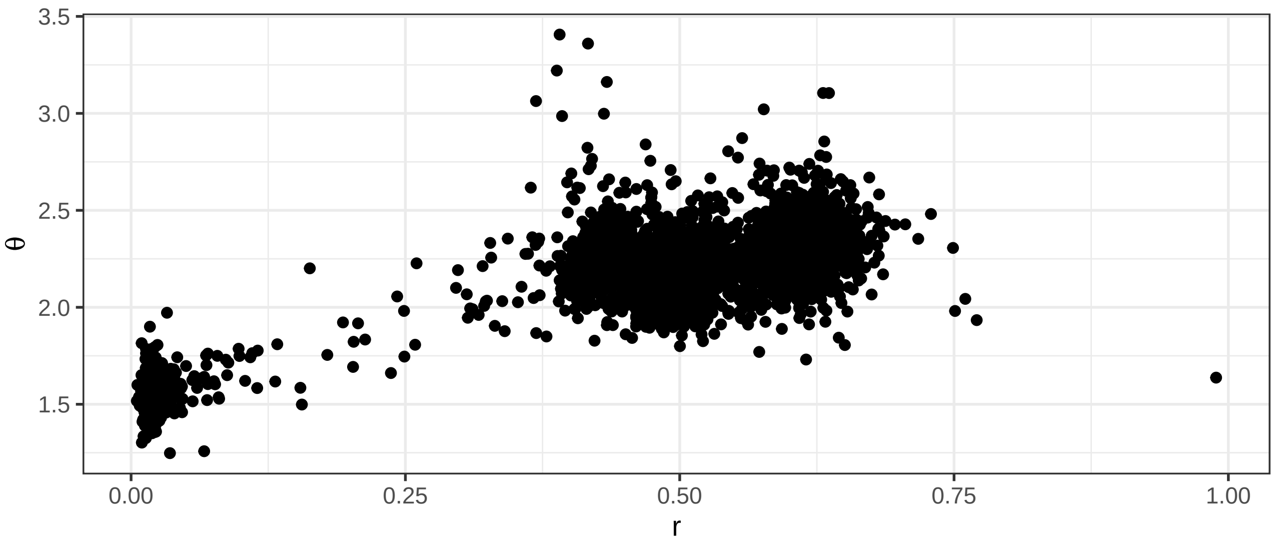

(a) Marker data, without a genetic map.

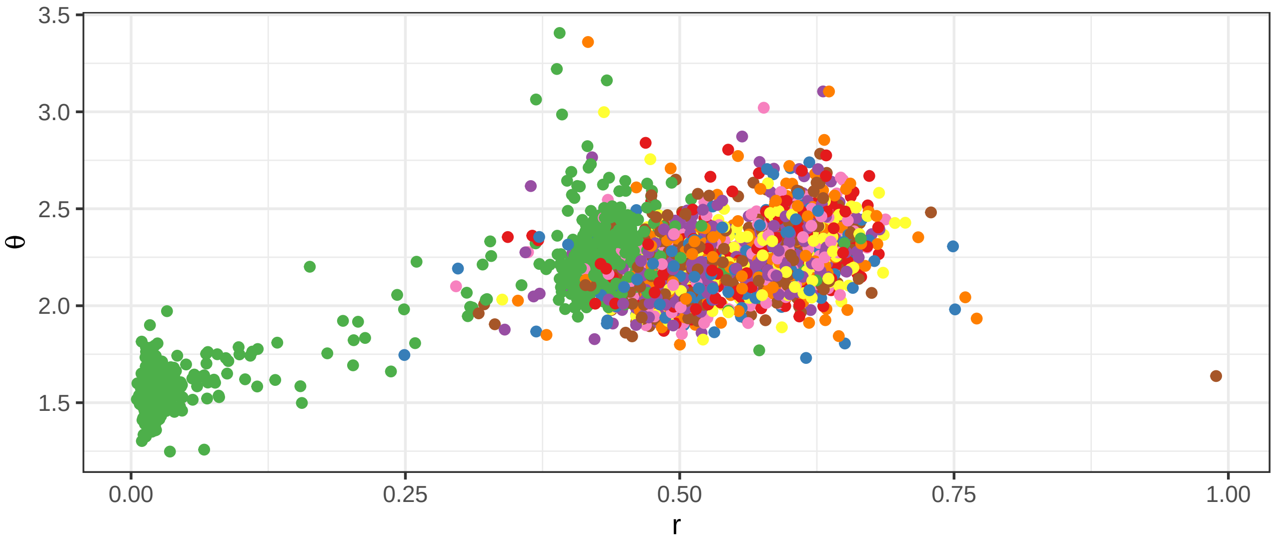

(b) Plot of the marker data, after a genetic map is constructed. Colour represents the final imputed founder genotype, at a location on chromosome 2B.

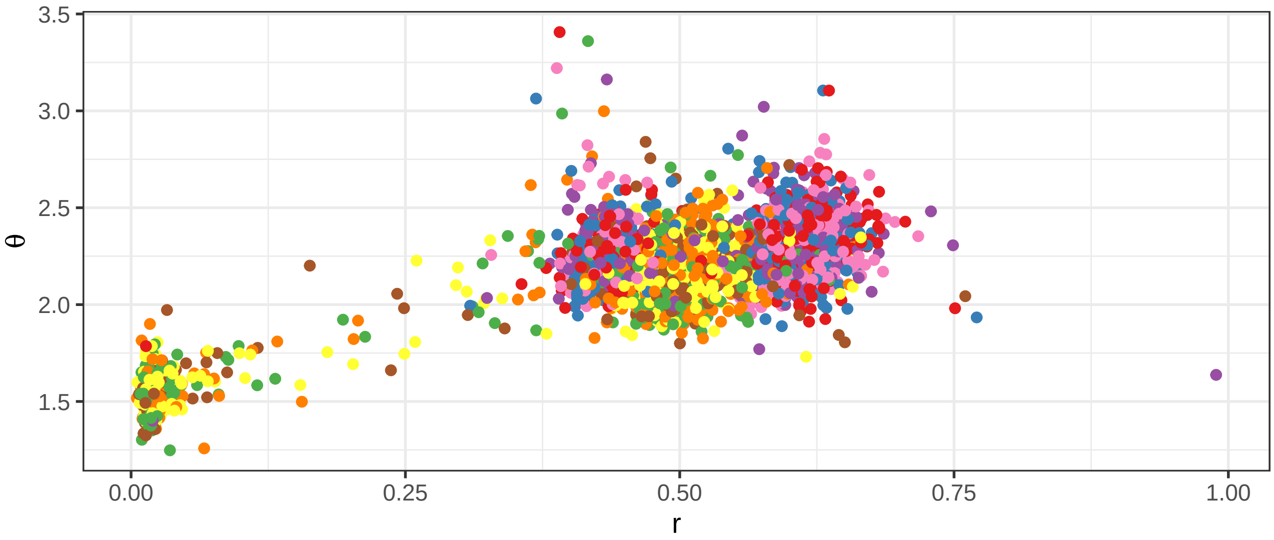

(c) Plot of the marker data, after a genetic map is constructed. Colour represents the final imputed founder genotype, at a location on chromosome 2A.

Figure S2: Example of a marker that is polymorphic on more than one chromosome. This marker (Kukri_c37840_253) is polymorphic on chromosomes 2A and 2B, although this is not obvious without a genetic map.

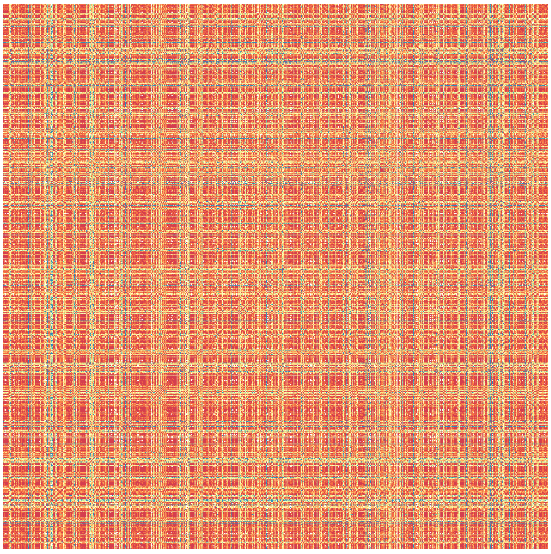

(a) Before ordering. Markers are in a random order.

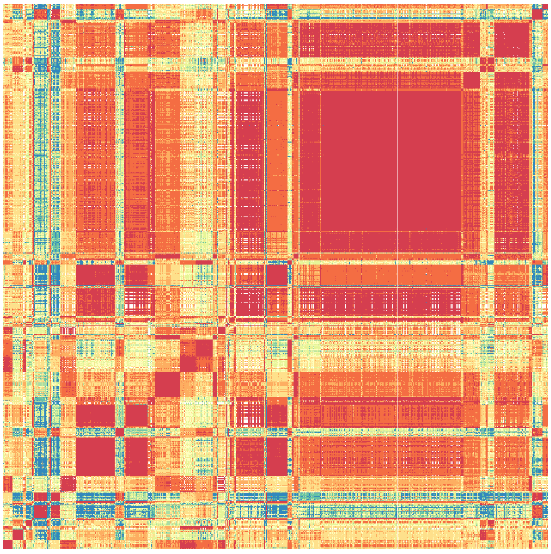

(b) After ordering the markers into 50 blocks. Markers within blocks are unordered.

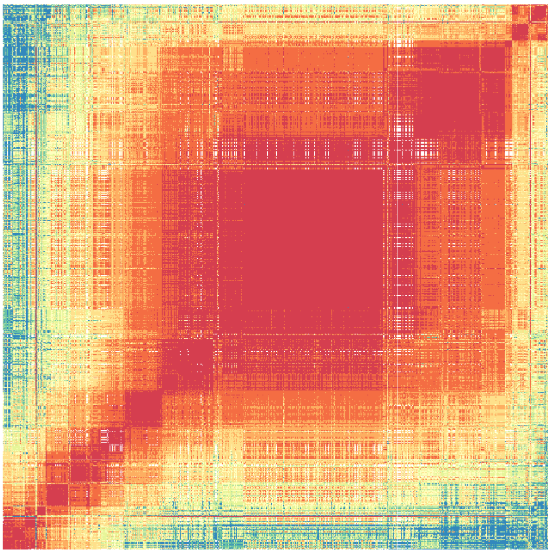

(c) After ordering the markers into 50 blocks, and then ordering the 50 blocks. Markers within blocks are still unordered.

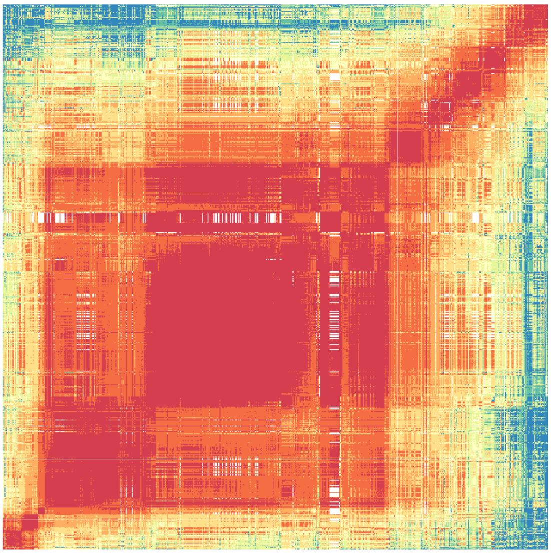

(d) A close to optimal ordering for this particular set of markers.

Figure S3: Rows and columns represent genetic markers on this chromosome, and values in the image represent estimated recombination fraction between a pair of markers. The ordering of the markers is different in all four subfigures. Red represents small recombination fractions, while blue represents large values. (a) The ordering of markers is initially random. (b) The markers are grouped into 50 large blocks. (c) These blocks are then ordered. (d) A close to optimal ordering.

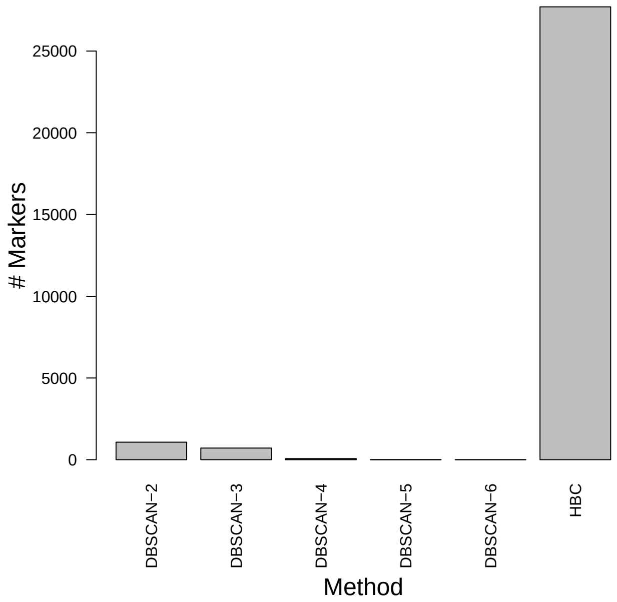

Figure S4: Number of times each genotype calling method was used. Method "DBSCAN-n" indicates the use of DBSCAN, where n is the number of alleles identified by DBSCAN.

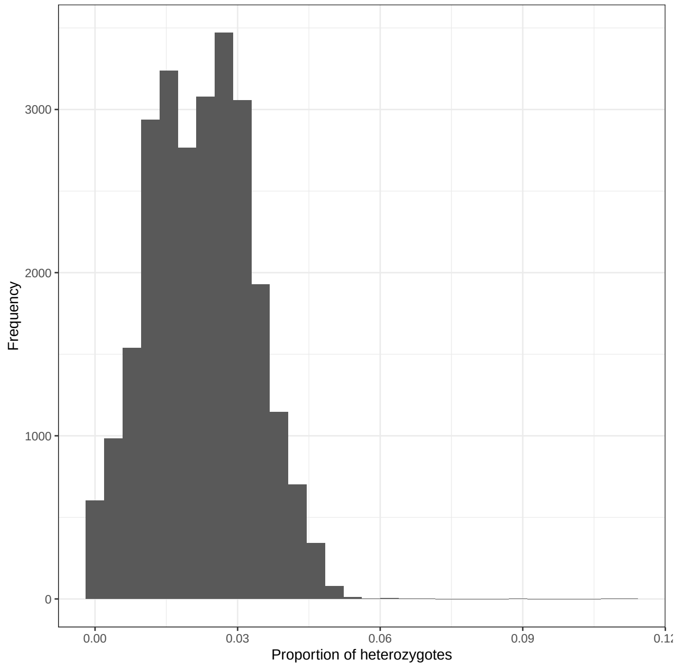

Figure S5: Distribution of the proportion of *marker* heterozygotes, for markers called using HBC. Note that not all identity-by-descent heterozygotes are marker heterozygotes.

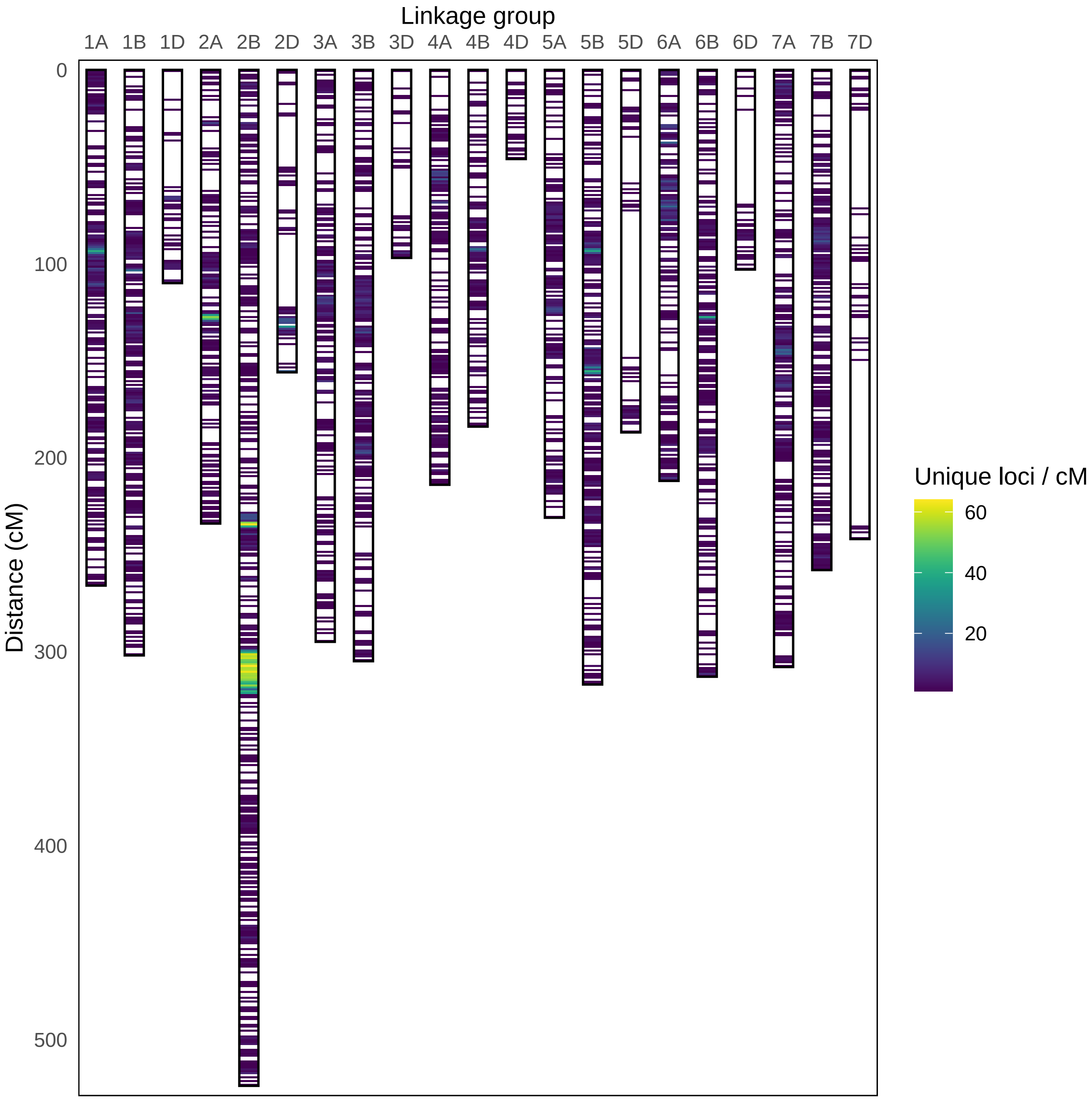

Figure S6: Illustration of the density of loci in the final map generated from the MAGIC population. White shows regions without map loci. Dark colour indicates fewer loci / cM, bright colour indicates more loci / cM.

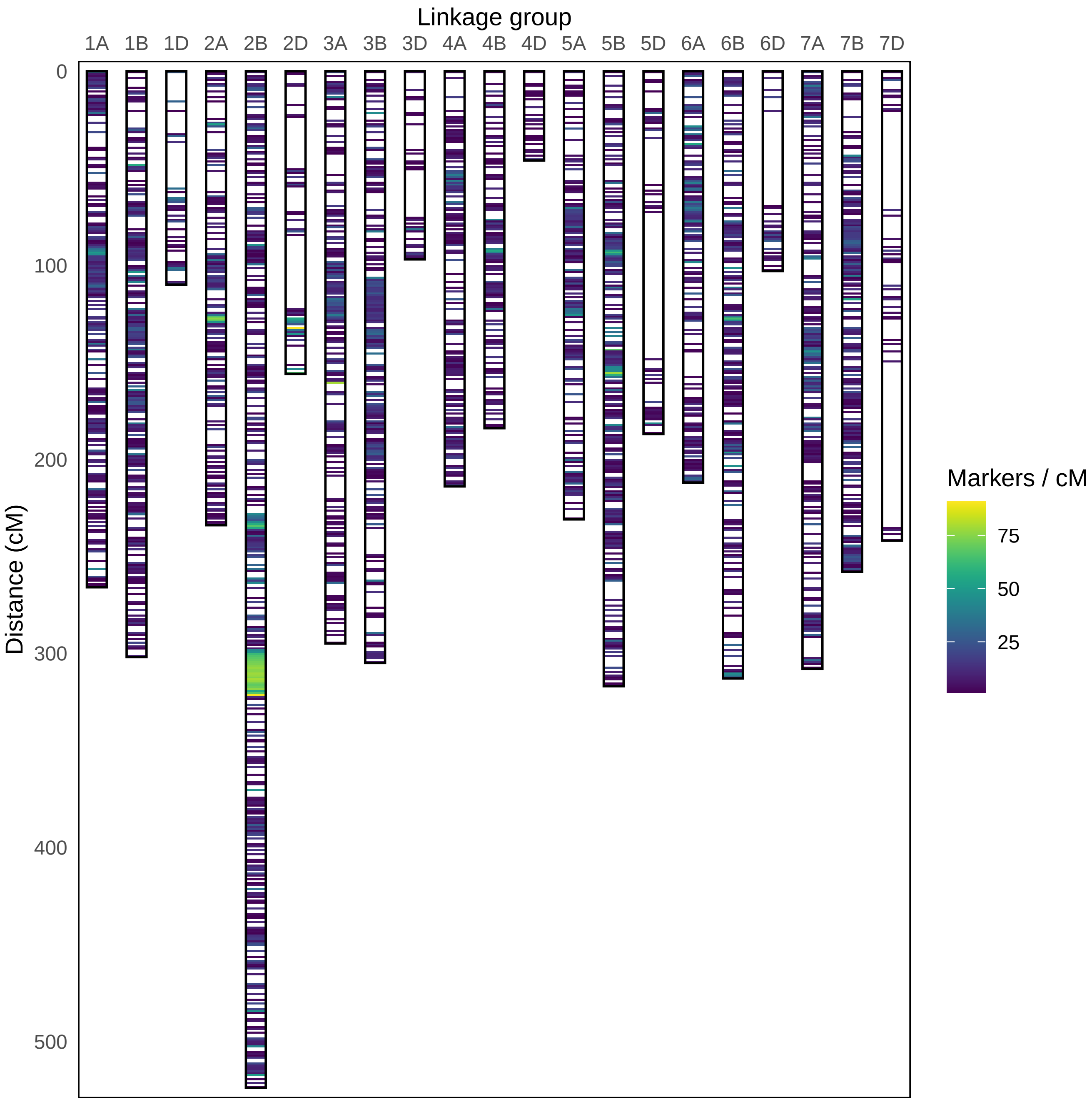

Figure S7: Illustration of the density of markers in the final map generated from the MAGIC population. White shows regions without marker coverage. Dark colour indicates low marker density, bright colour indicates high marker density.

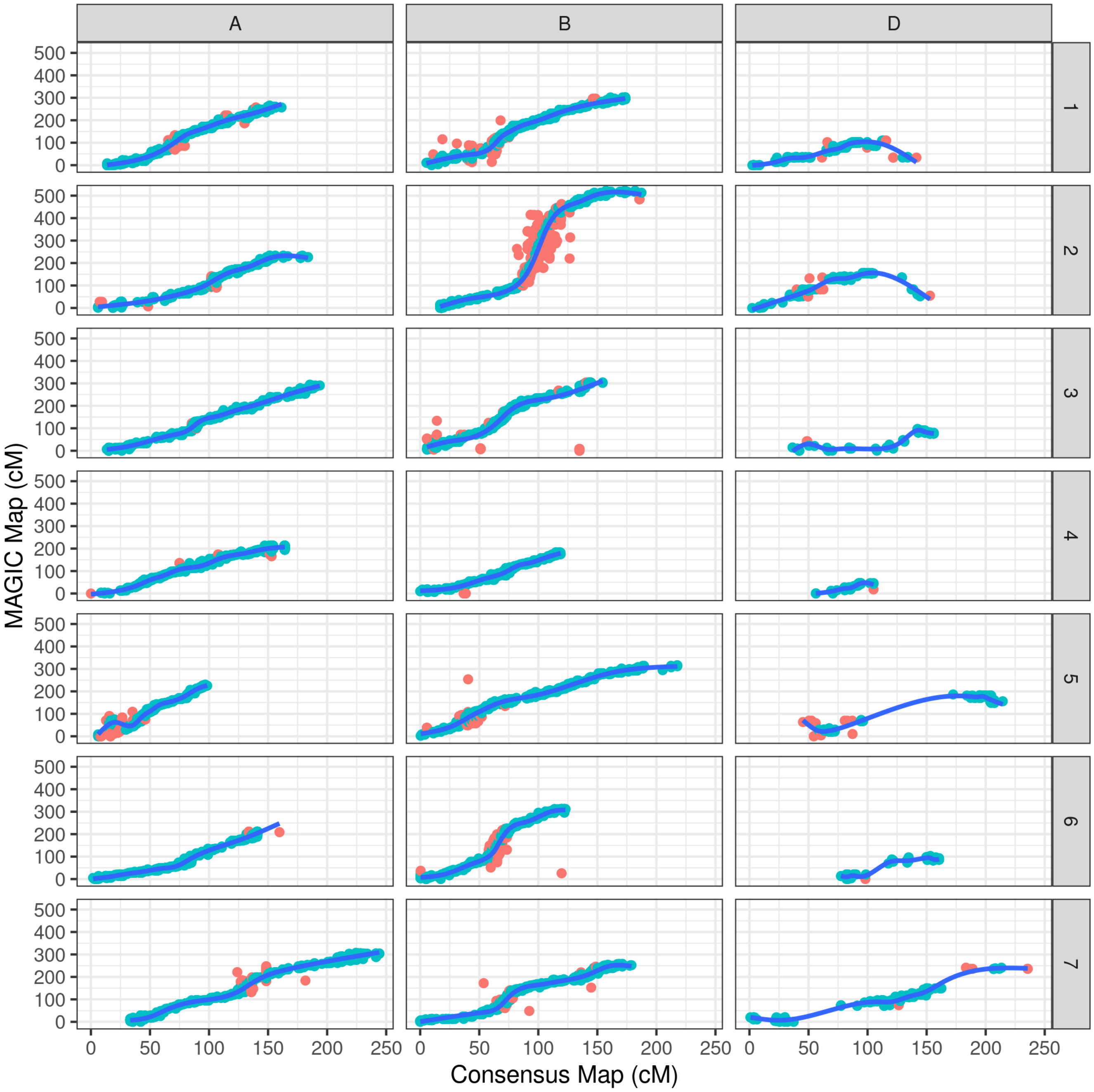

Figure S8: Comparison of the MAGIC map with the 90K consensus map. The blue line represents a best fit curve. Red points denote markers whoose positions deviate more than 20cM vertically from the curve.

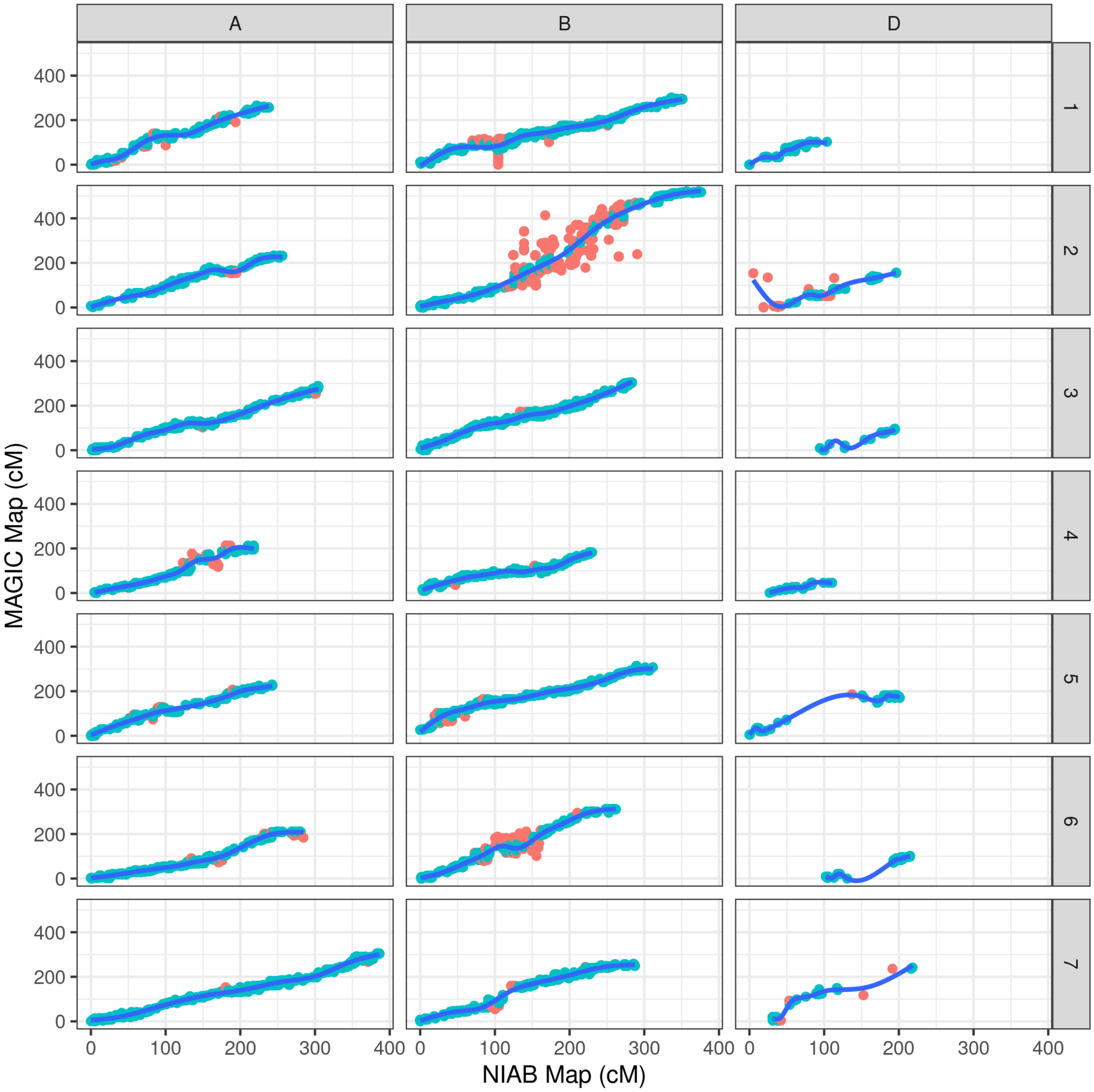

Figure S9: Comparison of the MAGIC map with the NIAB map. The blue line represents a best fit curve. Red points denote markers whoose positions deviate more than 20cM vertically from the curve.

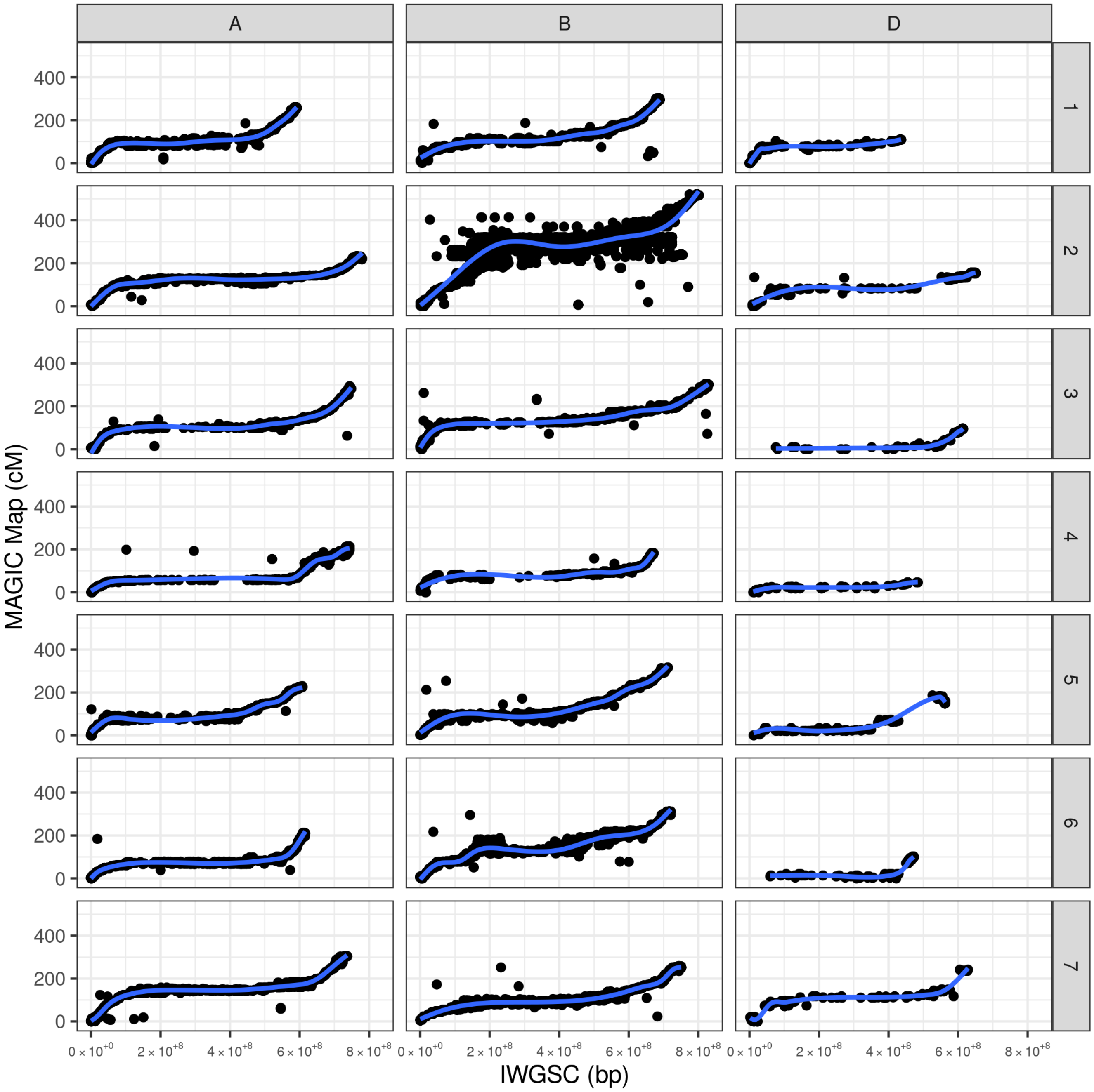

Figure S10: Comparison of the MAGIC map with the IWGSC RefSeq v1.0. The blue line represents a best fit curve.

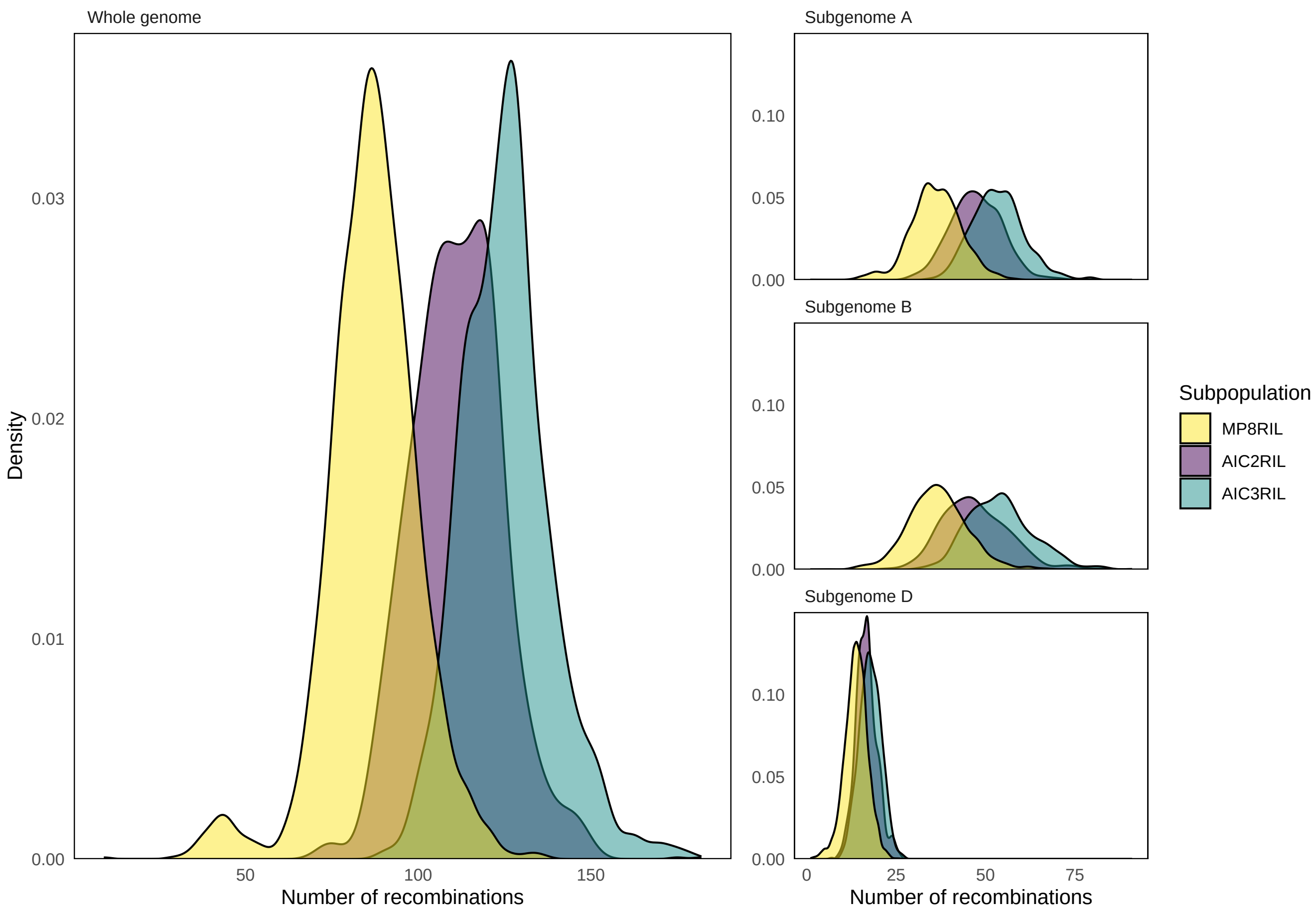

Figure S11: Distributions of the total number of recombination events for each subpopulation by, whole genome and subgenomes.

Chromosome	# Markers	# Distorted Markers	# Unique Positions	Length	Resolution
1A	1717	114	503	265.49	1.89
1B	1988	157	482	301.72	1.60
1D	393	66	84	109.85	0.76
2A	1376	520	389	233.54	1.67
2B	4899	3839	1886	523.63	3.60
2D	646	105	171	155.96	1.10
3A	1387	73	347	294.39	1.18
3B	1832	130	463	304.99	1.52
3D	148	5	46	96.88	0.47
4A	1059	83	269	213.08	1.26
4B	782	77	208	183.68	1.13
4D	79	15	25	45.93	0.54
5A	1311	238	321	230.25	1.39
5B	2392	210	634	316.14	2.01
5D	244	17	64	186.17	0.34
6A	1545	59	453	211.40	2.14
6B	1805	998	350	312.70	1.12
6D	222	22	36	102.27	0.35
7A	1854	408	485	307.20	1.58
7B	1804	107	416	257.62	1.61
7D	204	44	42	241.16	0.17
All	27687	7287	7674	4894.06	1.57

Table S2: Summary of the genetic map by chromosome, including number of markers mapped; number of markers with segregation distortion ($p < 10^{-5}$); length of chromosomes (centiMorgans); and resolution of the map, defined as the number of unique loci per centiMorgan.

		Consensus		NIAB	
Mapped	Chromosome	Common	Conflicts	Common	Conflicts
1717	1A	1464	40	604	22
1988	1B	1741	45	1180	107
393	1D	347	11	251	0
1376	2A	1168	48	540	15
4899	2B	2314	665	1066	396
646	2D	564	11	397	21
1387	3A	1161	2	723	4
1832	3B	1561	34	968	4
148	3D	109	3	71	0
1059	4A	938	7	347	34
782	4B	665	2	408	5
79	4D	63	1	47	0
1311	5A	1067	93	625	9
2392	5B	2087	67	1269	21
244	5D	165	13	154	1
1545	6A	1331	5	904	19
1805	6B	1542	202	889	133
222	6D	150	1	115	0
1854	7A	1490	101	1126	2
1804	7B	1582	30	609	25
204	7D	164	6	58	5

Table S3: Comparison of the MAGIC genetic map with the 90K consensus map and the NIAB map. For each chromosome, we present the number of markers mapped in our map (Mapped); the number of those in common with each comparison map (Common); and the number of disagreements (Conflicts) based on markers with distance >20 cM from the best fit curve to the comparison of map positions. Conflicts may be less accurate for D chromosomes as the smaller number of markers can lead to overfitting of the curve.

Chromosome	MP8RIL	AIC2RIL	AIC3RIL
1A	5.15	7.09	7.80
1B	5.26	6.75	8.10
1D	2.18	2.79	2.81
2A	5.01	6.71	7.37
2B	6.62	8.30	9.40
2D	2.64	3.05	3.37
3A	5.85	7.64	8.90
3B	5.67	7.42	8.40
3D	1.57	1.92	1.91
4A	5.03	6.61	7.46
4B	3.52	4.80	5.21
4D	0.77	1.02	1.16
5A	4.79	5.79	6.60
5B	6.93	8.87	10.39
5D	2.24	2.74	2.95
6A	4.19	5.35	6.15
6B	4.16	5.34	6.22
6D	1.42	1.69	1.80
7A	6.20	8.21	9.23
7B	4.59	5.76	6.56
7D	3.01	3.51	3.77
Whole Genome	86.82	111.36	125.57

Table S4: Average number of recombination events for each chromosome, for each of the three subpopulations.

Subpopulation	AC-Barrie	Alsen	Baxter	Pastor	Volcani	Westonia	Xiaoyan	Yitpi
All	0.19	0.12	0.11	0.07	0.18	0.07	0.15	0.11
MP8RIL	0.19	0.13	0.10	0.06	0.18	0.07	0.15	0.12
AIC2RIL	0.19	0.10	0.11	0.08	0.19	0.08	0.14	0.10
AIC3RIL	0.19	0.11	0.12	0.07	0.19	0.05	0.16	0.11

Table S5: Genetic composition at position 155 cM on chromosome 2D.

Subpopulation	AC-Barrie	Alsen	Baxter	Pastor	Volcani	Westonia	Xiaoyan	Yitpi
All	0.05	0.17	0.14	0.14	0.12	0.12	0.12	0.14
MP8RIL	0.06	0.16	0.13	0.13	0.12	0.12	0.13	0.13
AIC2RIL	0.06	0.21	0.13	0.12	0.11	0.11	0.12	0.13
AIC3RIL	0.03	0.18	0.15	0.16	0.12	0.13	0.08	0.15

Table S6: Genetic composition at position 136 cM on chromosome 4A.

Subpopulation	AC-Barrie	Alsen	Baxter	Pastor	Volcani	Westonia	Xiaoyan	Yitpi
All	0.09	0.22	0.17	0.11	0.16	0.08	0.09	0.09
MP8RIL	0.09	0.21	0.17	0.10	0.14	0.10	0.08	0.10
AIC2RIL	0.08	0.22	0.16	0.13	0.20	0.06	0.10	0.06
AIC3RIL	0.09	0.24	0.15	0.13	0.18	0.05	0.11	0.06

Table S7: Genetic composition at position 72 cM on chromosome 7D.

Subpopulation	AC-Barrie	Alsen	Baxter	Pastor	Volcani	Westonia	Xiaoyan	Yitpi
All	0.15	0.12	0.10	0.09	0.14	0.07	0.17	0.15
MP8RIL	0.14	0.12	0.10	0.09	0.14	0.07	0.18	0.15
AIC2RIL	0.14	0.09	0.15	0.10	0.14	0.07	0.17	0.14
AIC3RIL	0.17	0.15	0.11	0.08	0.14	0.06	0.14	0.16

Table S8: Genetic composition at position 238 cM on chromosome 7D.

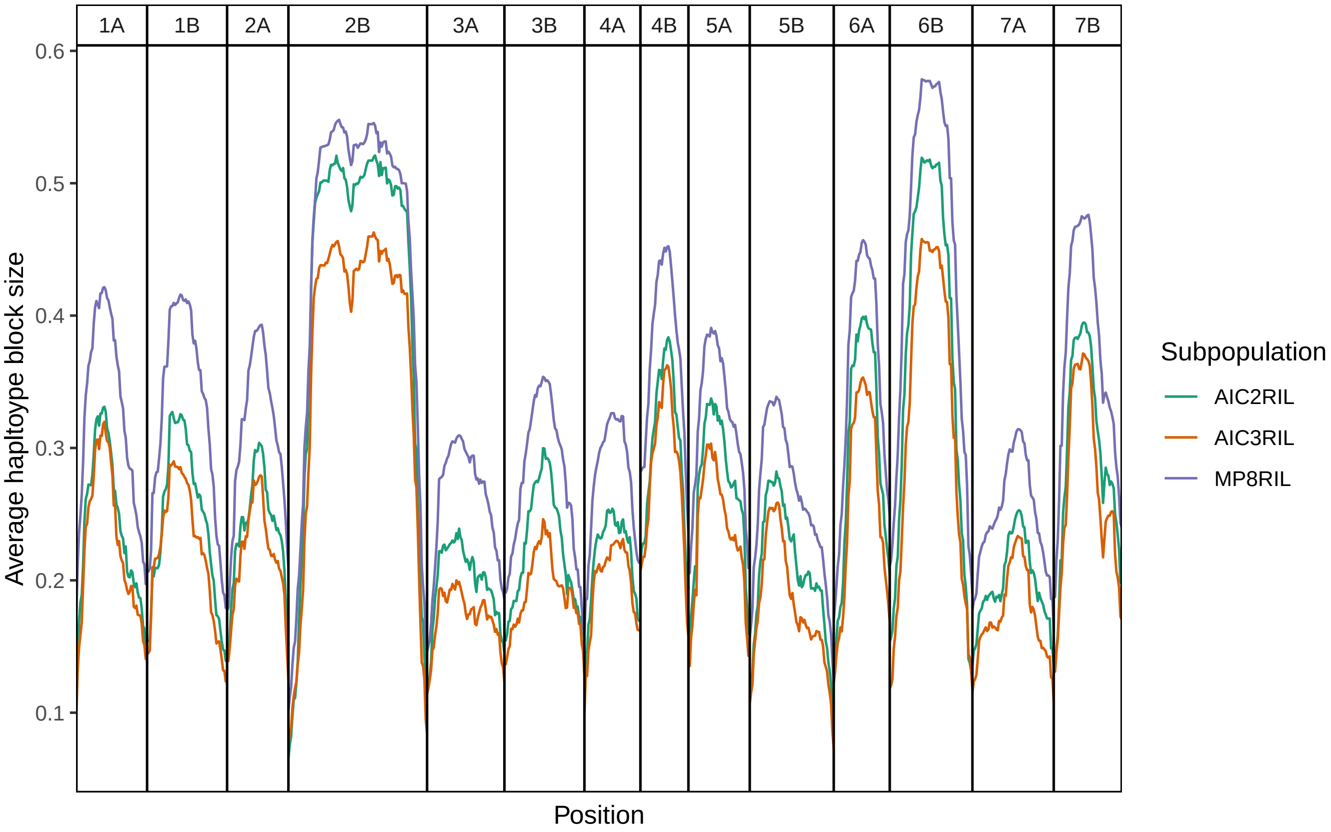

Figure S12: Average haplotype block sizes for the A and B genomes, as a proportion of the total chromosome length.

	AC-Barrie	Alsen	Baxter	Pastor	Volcani	Westonia	Xiaoyan	Yitpi	Total
AC-Barrie	0.01	0.01	0.02	0.01	0.01	0.02	0.01	0.01	0.09
Alsen	0.01	0.01	0.02	0.01	0.01	0.02	0.01	0.01	0.10
Baxter	0.02	0.01	0.05	0.01	0.02	0.05	0.01	0.05	0.23
Pastor	0.01	0.01	0.02	0.01	0.01	0.03	0.01	0.02	0.12
Volcani	0.01	0.02	0.02	0.02	0.01	0.02	0.01	0.02	0.13
Westonia	0.01	0.01	0.02	0.01	0.01	0.02	0.01	0.02	0.09
Xiaoyan	0.01	0.01	0.02	0.02	0.01	0.02	0.01	0.03	0.14
Yitpi	0.01	0.01	0.02	0.02	0.01	0.01	0.01	0.01	0.10
Total	0.09	0.10	0.19	0.09	0.09	0.19	0.07	0.18	

Table S9: Observed distribution of alleles, for the most significant 2B-6B interaction. Rows represent the genotype at position 462 cM on chromosome 2B. Columns represent the genotype at position 306 cM on chromosome 6B.

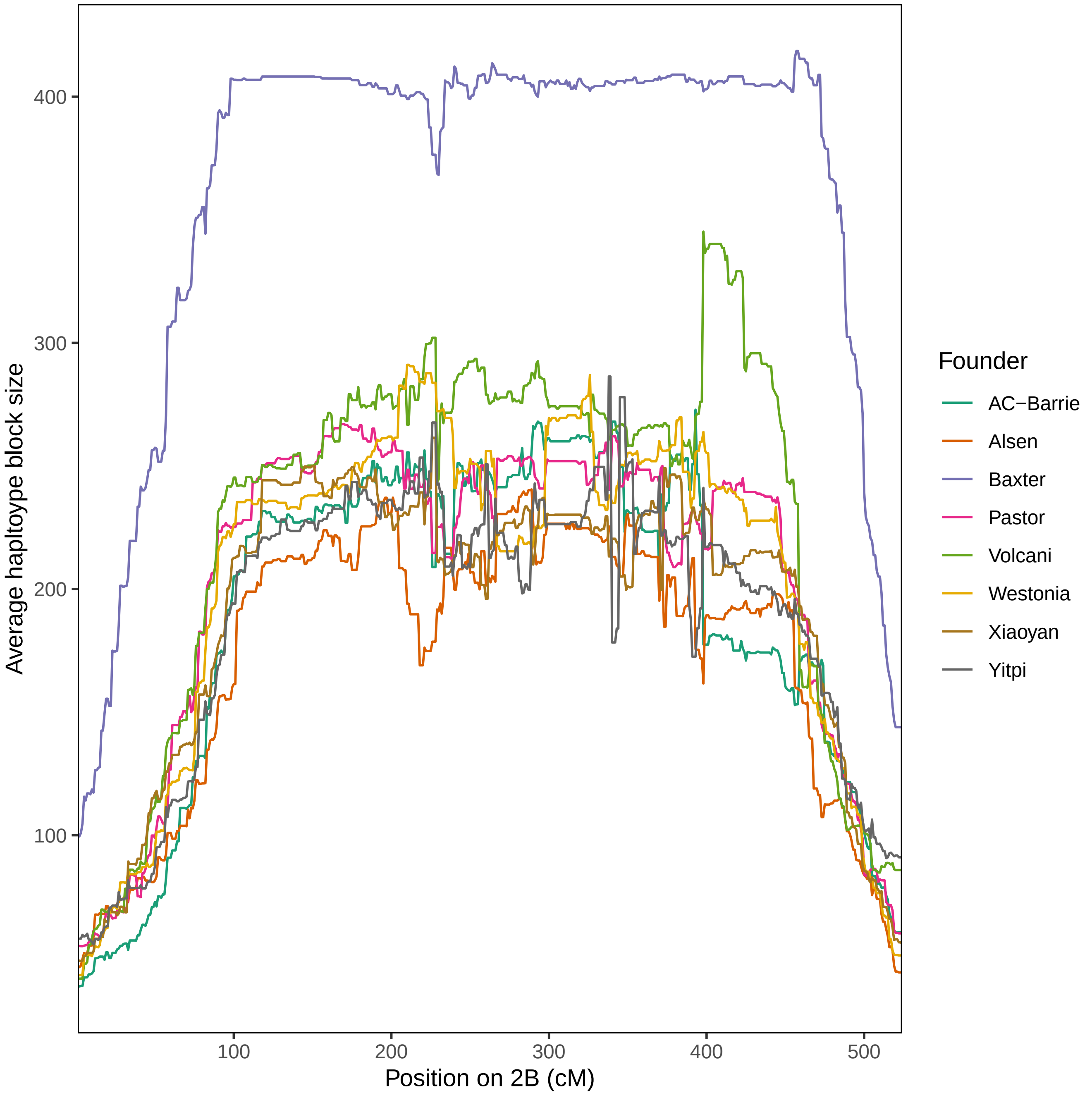

Figure S13: Average haplotype block sizes for each founder, at different positions on chromosome 2B.

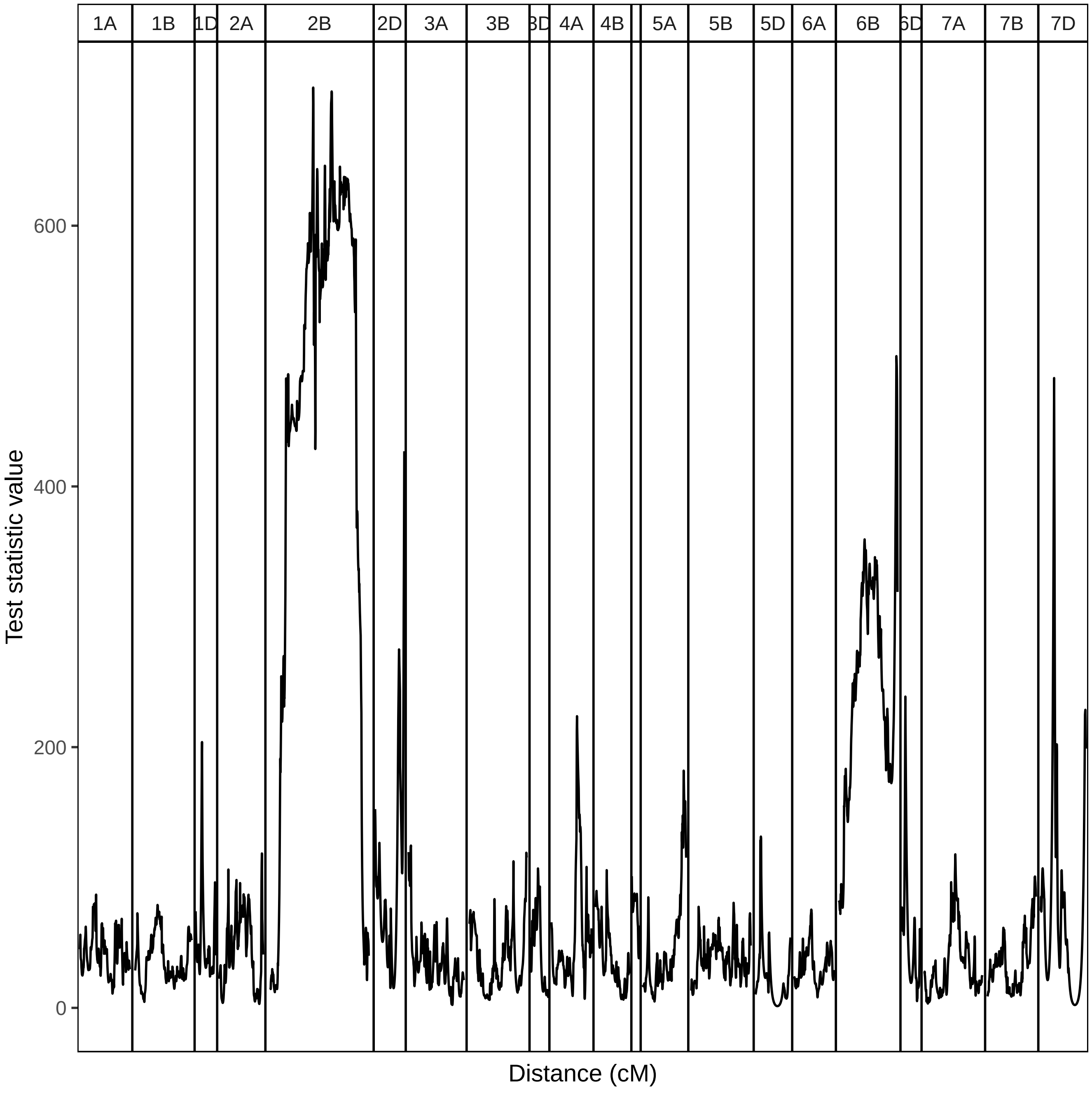

Figure S14: Chi-squared test statistic values, for tests of segregation distortion. Chromosome 4D is not labelled due to the width of the panel.

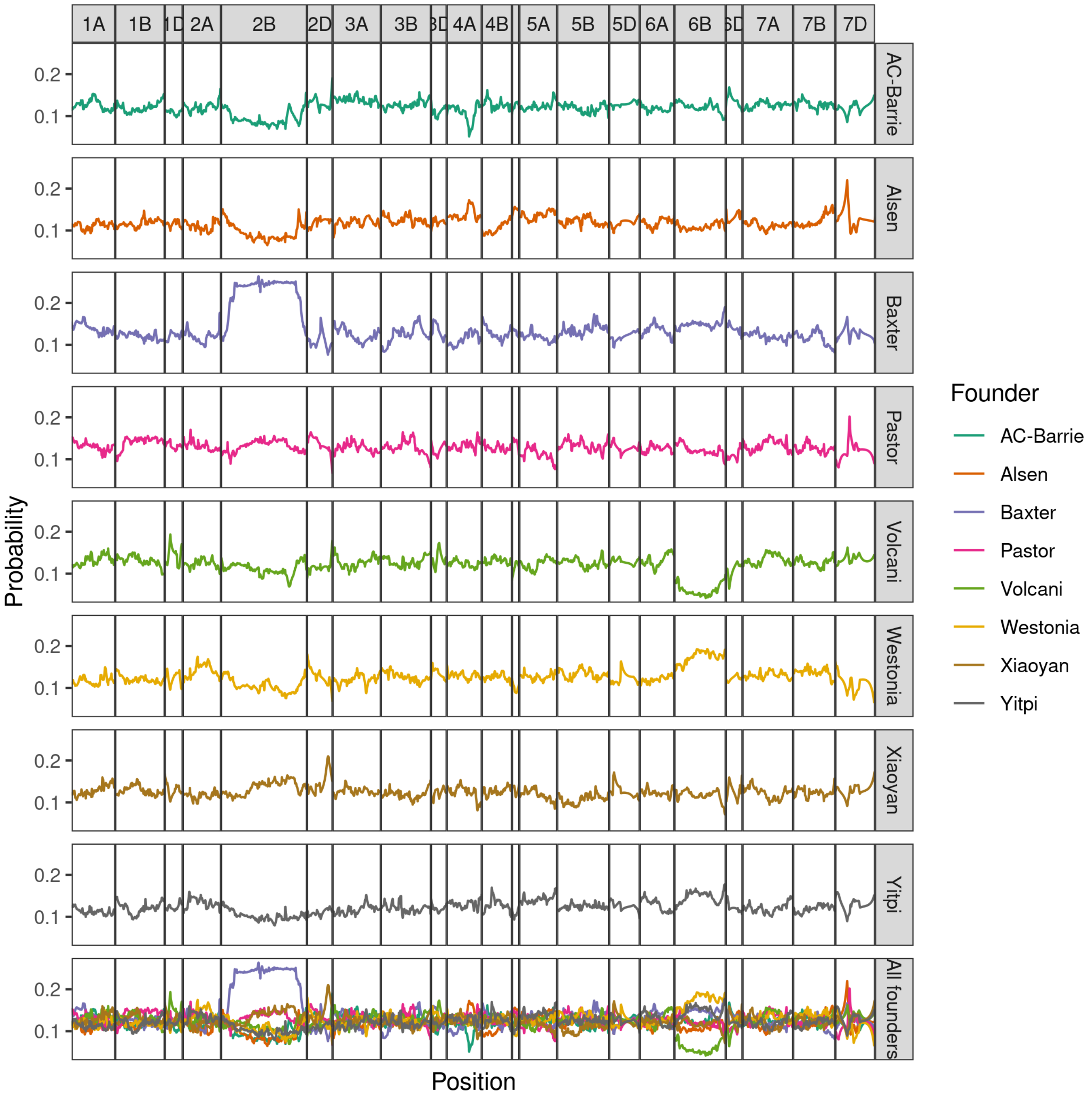

Figure S15: Average genetic composition across the entire population

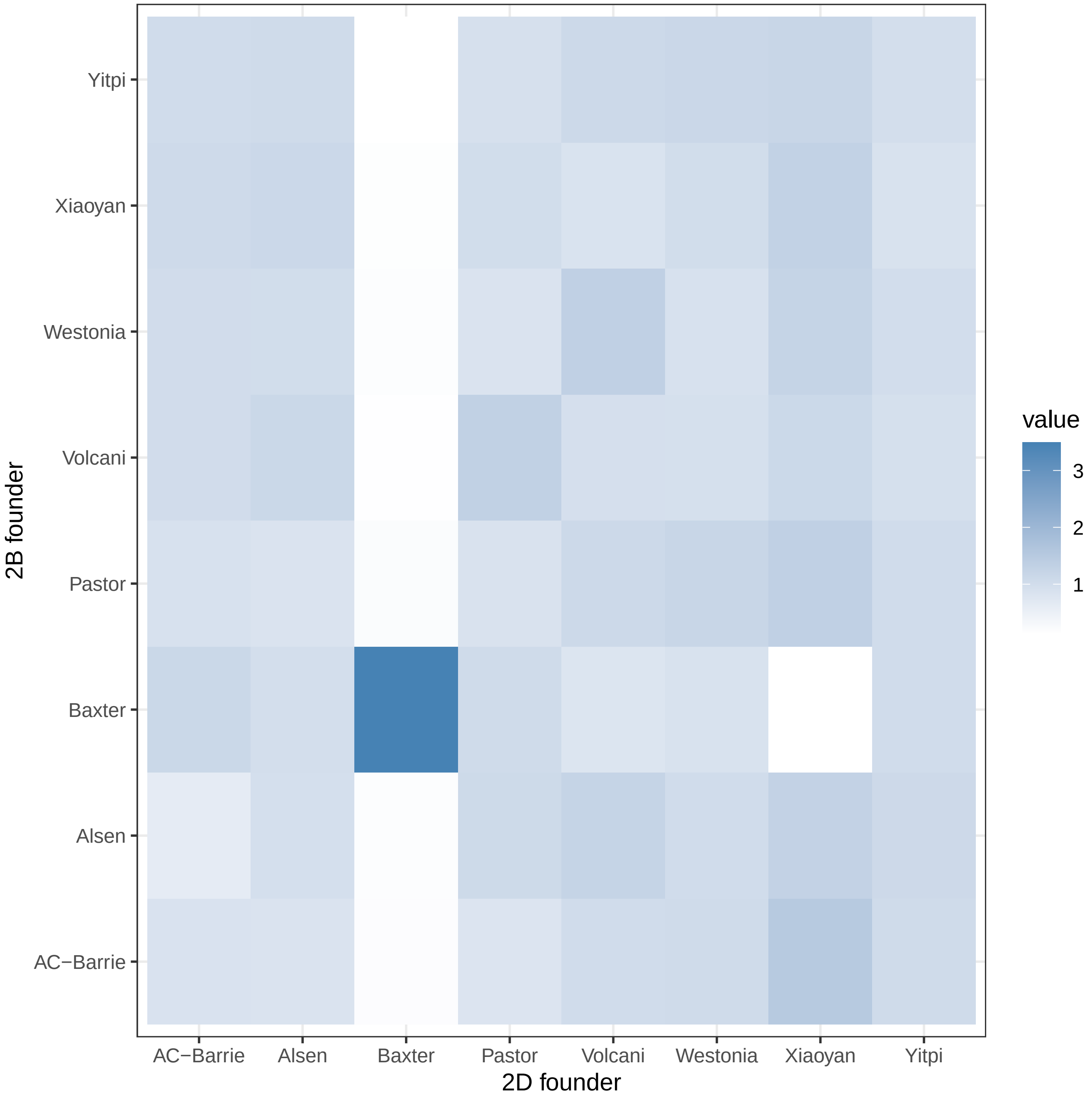

Figure S16: Visualisation of the 2B-2D interaction in Table 1. Colour represents the observed frequency as a multiple of the expected frequency under independence. Dark blue represents combinations present much more frequently than expected.

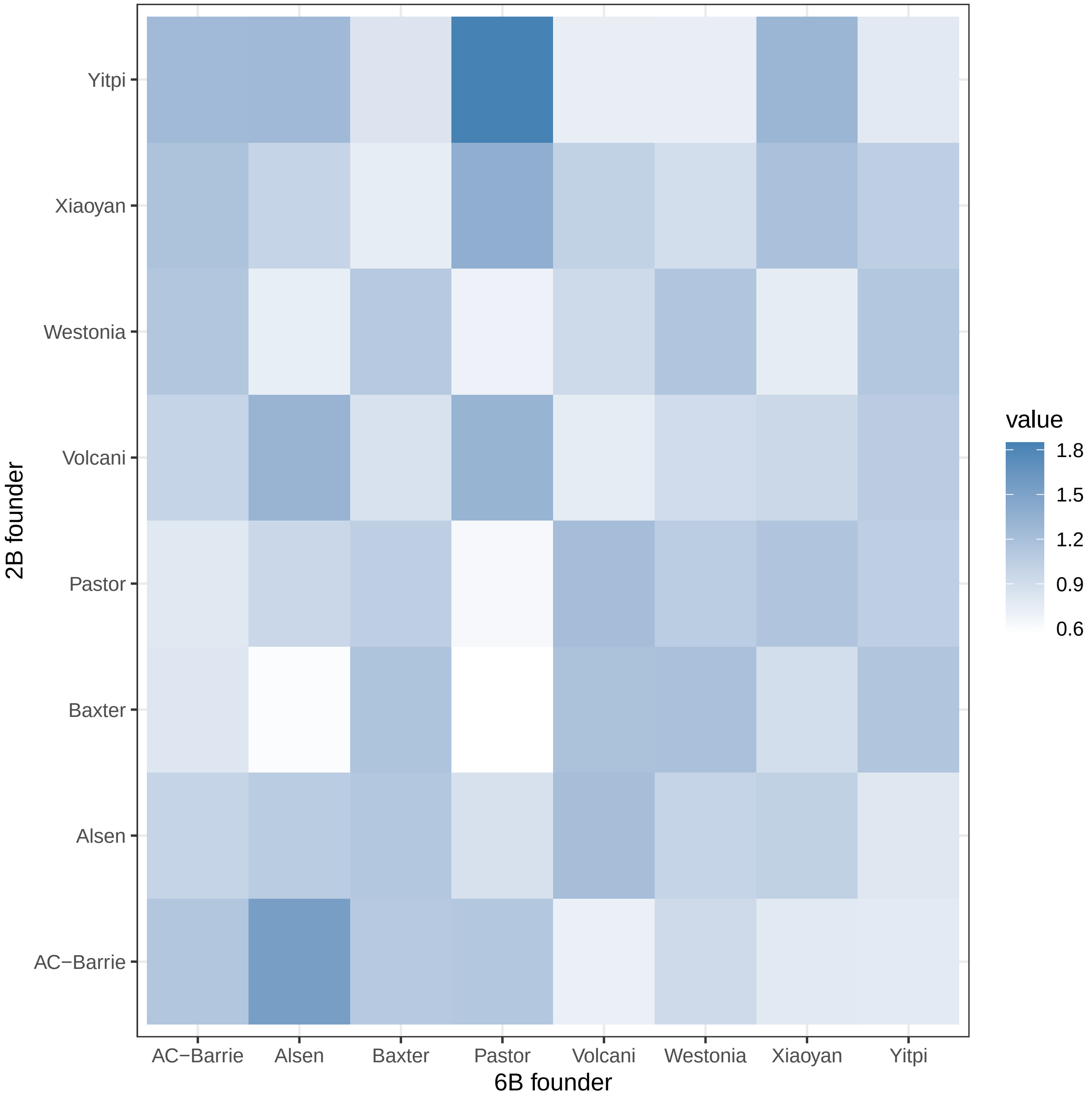

Figure S17: Visualisation of the 2B-6B interaction in Table 9. Colour represents the observed frequency as a multiple of the expected frequency under independence. Dark blue represents combinations present much more frequently than expected.

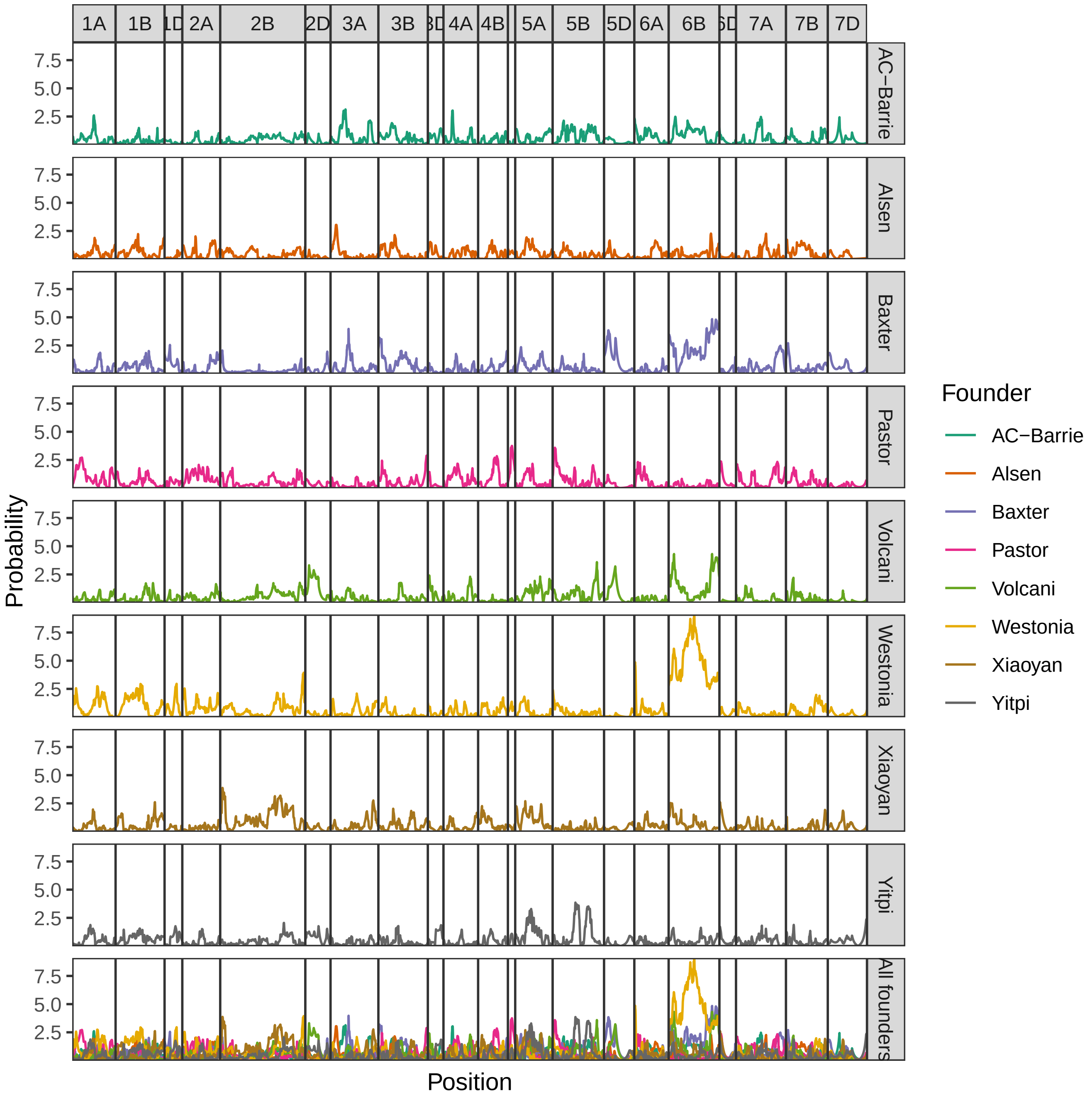

Figure S18: Chi-squared statistics for sex-specific segregation distortion.

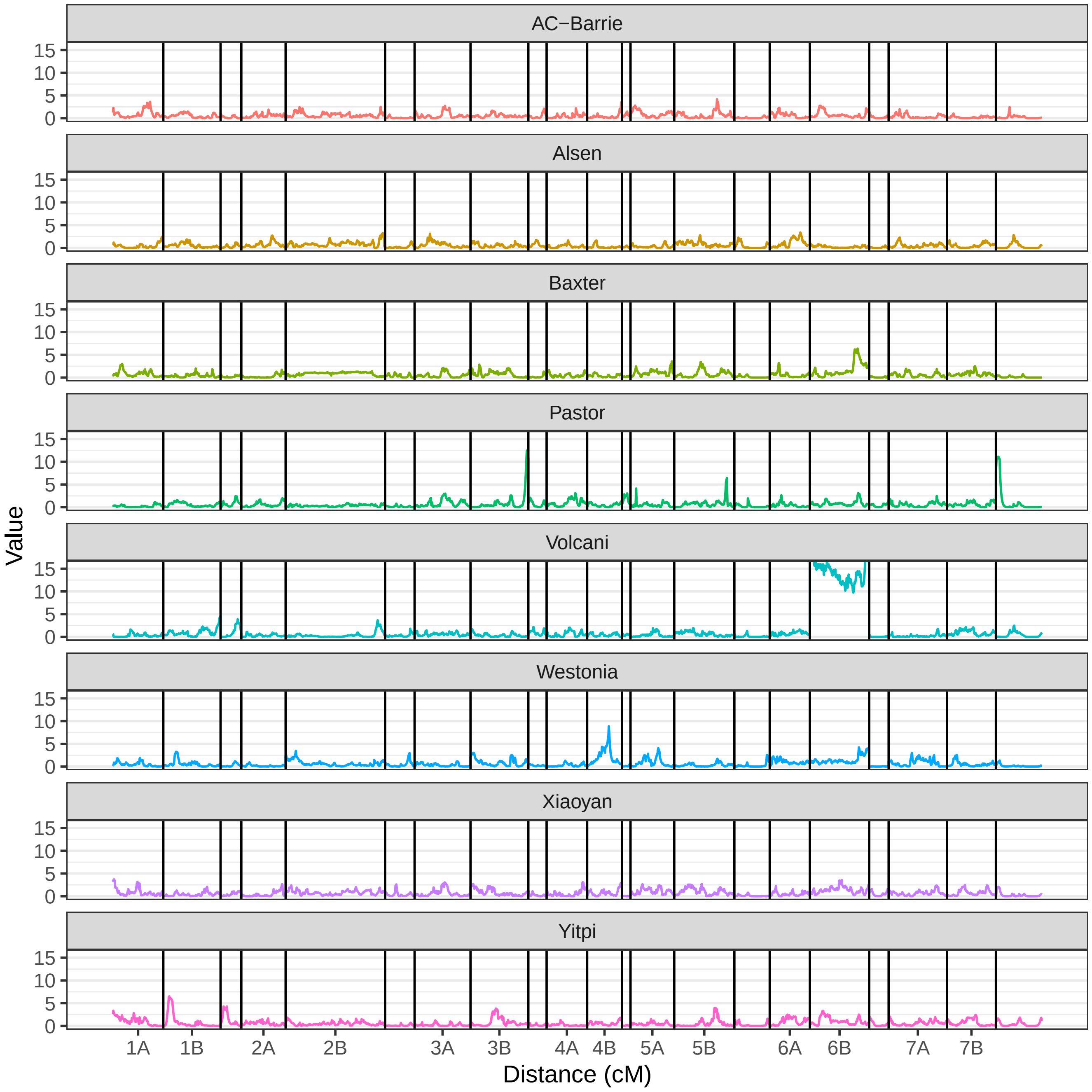

Figure S19: Chi-squared statistics for segregation distortion related to the crossing of founders in the first generation.

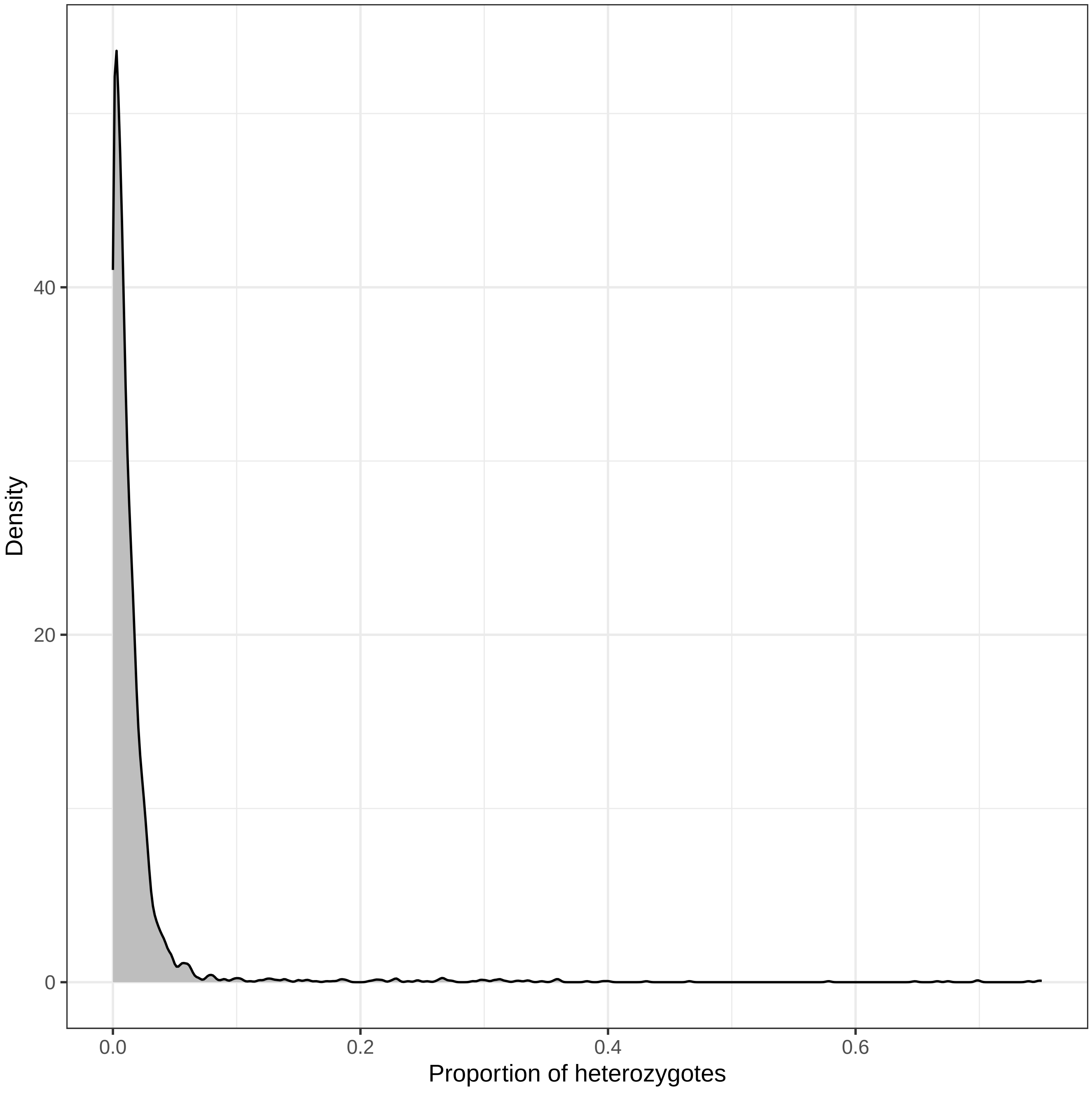

Figure S20: Distribution of the proportion of residual heterozygosity, for all three subpopulations.
